# Chemical Profiling of Aboveground and Underground Parts of *Pterocephalus hookeri* by Integrated FBMN, Untargeted LC-MS Metabolomics, and PAD-DESI-MSI

**DOI:** 10.3390/molecules31111868

**Published:** 2026-05-29

**Authors:** Jiaxing Luo, Lanlan Fang, Muze Yu, Di Yang, Jing Zhang, Jia Yu, Ce Tang, Tingting Kuang

**Affiliations:** 1 Laboratory for Innovation & Effective Uses of Chinese Drug Germplasm Resources, School of Ethnic Medicine, Chengdu University of Traditional Chinese Medicine, Chengdu 611137, China; luojiaxing0624@163.com (J.L.); 13619090682@163.com (M.Y.); zhangjing1@cdutcm.edu.cn (J.Z.); yu77@cdutcm.edu.cn (J.Y.); tangce@cdutcm.edu.cn (C.T.); 2 School of Pharmacy, Chengdu University of Traditional Chinese Medicine, Chengdu 611137, China; fanglanlan@stu.cdutcm.edu.cn (L.F.); 18285699822@163.com (D.Y.)

**Keywords:** *Pterocephalus hookeri*, untargeted metabolomics, feature-based molecular networking, differential markers, UPLC-Q-TOF/MS, different medicinal parts, DESI-MSI

## Abstract

*Pterocephalus hookeri* (C.B.Clarke) Höeck is a classic traditional Tibetan medicinal herb with multiple pharmacological activities. The inconsistent usage of its medicinal parts (whole herb, aboveground part (AP), and underground part (UP)) in commercial circulation severely restricts its clinical safety and quality stability. Currently, most existing chemical investigations focus on the whole herb, whereas the intraspecific chemical discrepancies between AP and UP remain poorly clarified. Herein, an integrated analytical strategy combining ultra-high-performance liquid chromatography–quadrupole time-of-flight mass spectrometry (UPLC-Q-TOF/MS)-based untargeted metabolomics, feature-based molecular networking (FBMN), and paper-based analytical device desorption electrospray ionization mass spectrometry imaging (PAD-DESI-MSI) was established to characterize differential metabolites and their spatial distribution in *P. hookeri*. A total of 101 compounds were annotated, and 12 vital differential metabolites were further screened with variable importance in projection (VIP) values > 1. The visualized distribution differences of these biomarkers were validated via heatmap and PAD-DESI-MSI analysis. Obvious differences in chemical accumulation characteristics were confirmed between AP and UP, which can guide reasonable clinical medication and rational dosage regulation referring to metabolite abundance. Moreover, optimized data filtering thresholds effectively eliminated metabolomic false positives, and FBMN exhibited excellent capacity for differential biomarker screening. This study provides a solid chemical basis for the quality evaluation and rational medicinal application of *P. hookeri*.

## 1. Introduction

*Pterocephalus hookeri* (C.B.Clarke) Höeck is a classic medicinal herb in traditional Tibetan medicine with the effects of removing toxins to eliminate warm epidemic disease, clearing heat to stop dysentery, and dispelling wind to unblock impediments [1]. Clinically, it is frequently used to treat rheumatoid arthritis (RA) [2]. While the Chinese Pharmacopoeia 2025 (ChP 2025) allows the use of the entire plant as a medicinal material, products commonly available in the market are primarily derived from the UP. However, research indicates that the total concentration of oleanolic acid and ursolic acid in the UP of *P. hookeri* is below 0.20%, falling short of the ChP 2025 standards [3]. Furthermore, the over-exploitation of its UP has resulted in a remarkable reduction in *P. hookeri*’s wild resources, compromising the quality of these resources. Concurrently, the underutilization of the AP exacerbates resource wastage. Therefore, thoroughly investigating the chemical composition of *P. hookeri*’s various parts and optimizing the selection of its medicinal components are crucial for effectively augmenting the supply of medicinal materials, increasing their utilization, and ensuring sustainable resource use. This approach not only minimizes resource wastage but also improves the quality and efficacy of the medicinal materials.

Metabolomics, a fundamental component of systems biology, functions to precisely identify the types and concentrations of metabolites at specific timepoints under defined conditions [4]. This discipline is characterized by its holistic, dynamic, and comprehensive nature, aligning closely with the “multi-component, multi-target, multi-pathway” nature of traditional Chinese medicine [5]. Thus, it serves as an effective tool for analyzing the efficacy and mechanisms of traditional Chinese medicine [6]. As a vital branch, plant metabolomics utilizes chemometric techniques for qualitative and quantitative analyses of total plant metabolites. This approach precisely distinguishes different sections of medicinal plants and identifies differential compounds by using multivariate statistical methods, thereby offering a robust scientific foundation for determining medicinal components and developing other plant parts.

Liquid chromatography–mass spectrometry (LC-MS), favored for its speed, high sensitivity, and superior structural elucidation abilities, has become the technology of choice for metabolomics research [7]. LC-MS not only delivers detailed structural information on compounds, such as molecular ion and ion fragment peaks, but also reveals fragmentation characteristics of the mass spectrum due to the actual metabolites, reagent impurities, and instrument residues in samples [8]. Accurate preprocessing of raw MS data is essential to extract dependable metabolite features and eliminate random errors and other interferences, ensuring data analysis accuracy and thorough information mining. Currently, the preprocessing of MS data involves critical steps such as peak extraction, alignment, noise filtering, and normalization. However, MS analysis may yield low response values for some low-abundance compounds. Such variables can distort subsequent multivariate statistical models and lead to false positives in screening results, particularly with variable importance in Projection (VIP) values greater than 1 [9,10]. Filtering raw MS data by response values can reduce the occurrence of such false positives.

The Global Natural Product Social (GNPS) Molecular Networking is a platform that visualizes molecular networks on the basis of MS data [11,12]. By utilizing secondary MS, this platform clusters molecules with analogous data to form a molecular network. Feature-based molecular networking (FBMN) improves upon classical molecular networking (CLMN) by integrating MS^1^-level information, including isotopic peaks and retention times (RTs), with ion intensity to subdivide molecules [13]. FBMN can distinguish isomers with similar secondary spectra and integrates relative quantitative data, supporting downstream statistical analysis of metabolites. By inputting multiple batches of sample data into FBMN simultaneously, FBMN generates a molecular network with colorful pie chart nodes. The sector areas of these pie chart nodes reflect the relative abundance of each compound across different samples [14]. Nodes exhibiting considerable compound abundance variations between samples may reveal crucial distributional differences. Thus, FBMN offers a strategic approach for identifying compounds with substantial differences.

Desorption electrospray ionization–mass spectrometry imaging (DESI-MSI) is an advanced analytical technique for investigating the spatial distribution of biomolecules in biological tissues. This technique enables direct analysis of biological tissue sections, providing high-throughput, comprehensive characterization of tissue composition through two-dimensional ion density maps that reveal compound distribution and relative abundance. These capabilities facilitate the elucidation of biological processes and biomarker dynamics [15]. The paper-based analytical device (PAD) represents a cost-effective, environmentally friendly detection method that utilizes capillary action, eliminating the need for external pumping systems. Conventional DESI-MSI analysis requires thin-sectioning of samples prior to analysis. For samples that are difficult to section, we developed an integrated paper-based analytical device coupled with desorption electrospray ionization mass spectrometry imaging (PAD-DESI-MSI) approach for spatial visualization [16].

**Figure 1:**
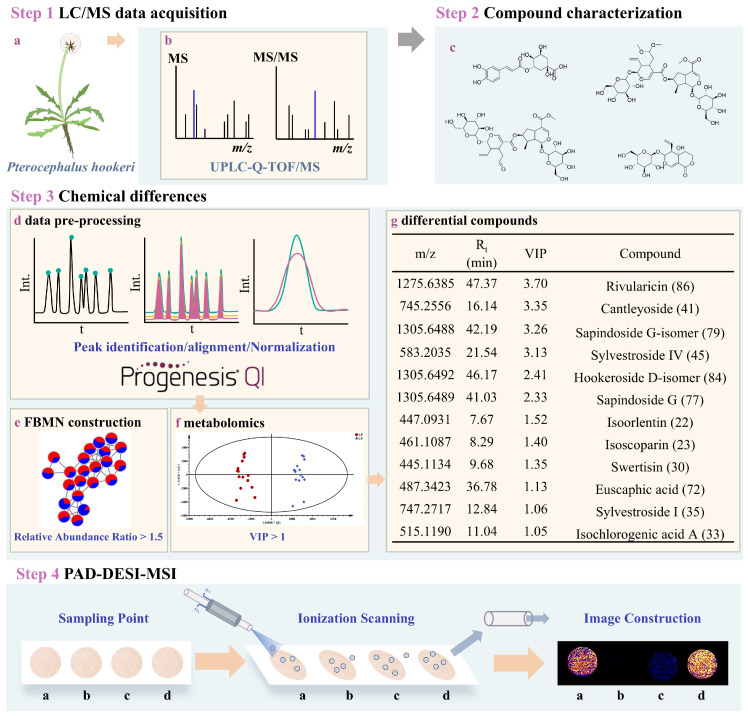
Research workflow of this study.

In this study, UPLC-Q-TOF/MS, metabolomics and FBMN were integrated to systematically characterize the chemical variability among AP and UP of *P. hookeri*. UPLC-Q-TOF/MS was used firstly to complete the qualitative analysis of chemical components, followed by metabolomics combined with FBMN technology to screen the differential components. By optimizing the mass spectrometry response threshold, an effective strategy to reduce the false-positive rate of metabolomics was established in this study. Based on hierarchical cluster analysis (HCA) heatmap with PAD-DESI-MSI spatial distribution imaging, the visual characterization of differential components was achieved. The results clarified the distribution pattern of differential components in AP and UP of *P. hookeri*, established the scientific basis for its quality control, and provided an innovative methodological reference for the screening of differential compounds in ethnic medicine. The research workflow of this work was summarized in Figure 1.

## 2. Results

In this study, 70% methanol extracts of *P. hookeri* were used as the research material for chemical component identification and differential metabolite analysis. The whole plant was used for preliminary component profiling, while the AP and UP were separately applied to screen and compare characteristic differential compounds.

### 2.1. Identification of Chemical Components of P. hookeri

**Figure 2:**
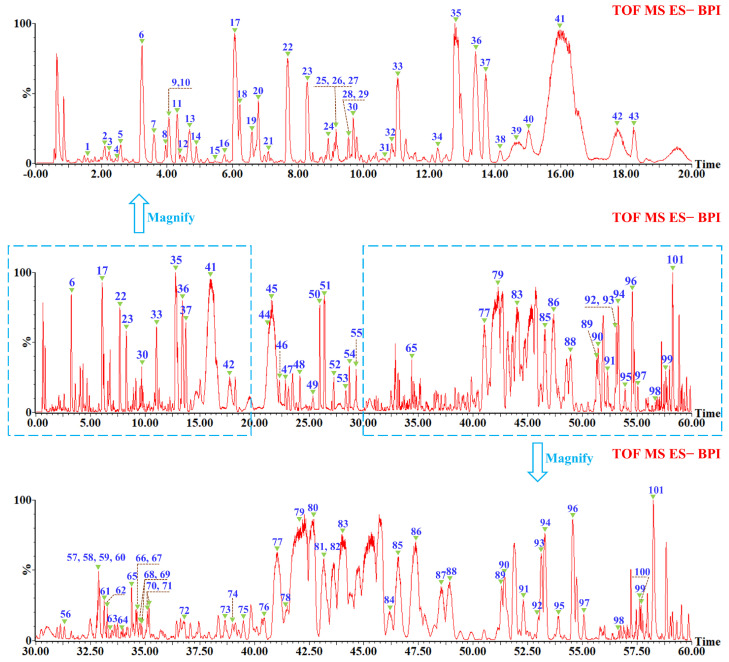
The representative base peak ion (BPI) chromatogram of *P. hookeri* in negative ion mode. The box and arrow denote the magnified section.

In this study, the primary chemical components of *P. hookeri* were rapidly separated and identified using UPLC-Q-TOF/MS. The samples were rapidly separated over a period of 60 min by using UPLC, which eluted at an optimal gradient mobile phase. Figure 2 displays the base peak ion chromatogram of *P. hookeri* in negative ion mode. Figure S1 shows the corresponding chromatogram in positive ion mode. A total of 101 components of *P. hookeri* (Figures S2–S4) were identified by correlating the reference substances, RTs, and literary sources with the quasi-molecular ions from the primary mass spectrum and the fragment ions from the secondary mass spectrum. Examples of these constituents include iridoids, phenylpropanoids, triterpenes, among other chemicals. Table 1 lists the precise molecular mass and ion fragmentation data for the identified compounds in negative ion mode. Table S1 lists the corresponding information in the positive ion mode. All identified compounds exhibited molecular ion mass errors below ±5 ppm, demonstrating high consistency between theoretical and experimental results.

#### 2.1.1. Iridoids

**Figure 3:**
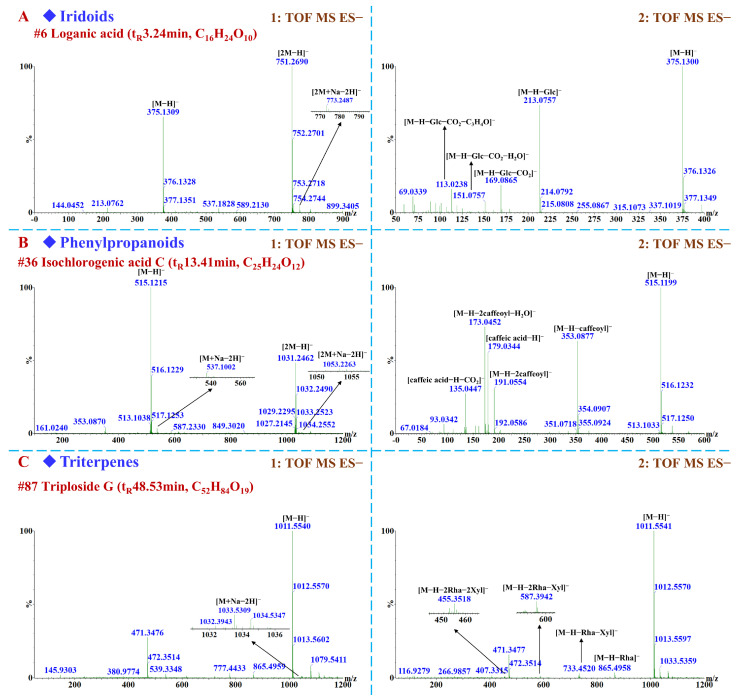
Primary and secondary mass spectrum of three major components in *P. hookeri*. (**A**) Loganic acid; (**B**) Isochlorogenic acid C; (**C**) Triploside G.

**Figure 4:**
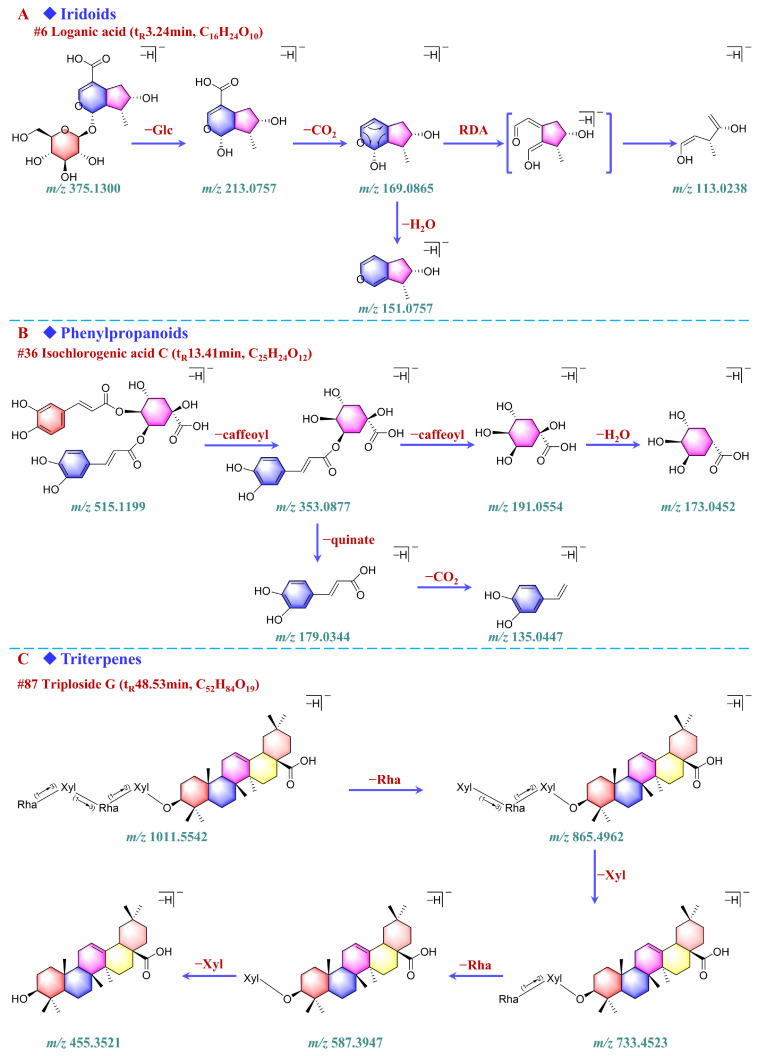
The cleavage pathway of three major components in *P. hookeri*. (**A**) Loganic acid; (**B**) Isochlorogenic acid C; (**C**) Triploside G.

In the mass spectrum, iridoids primarily undergo cleavage of their glycosidic bonds. A characteristic feature is the neutral loss of glucose moiety (Glc, 162 Da). Additionally, these compounds tend to lose neutral groups such as CO, CO_2_, H_2_O, and OCH_3_ [17]. Additive iridoid ions, including [M–H]^−^, and [M+Na–2H]^−^, are easily formed in the *P. hookeri* samples scanned in negative ion mode. A total of 24 iridoids were identified by analyzing fragment ion data in positive and negative ion modes. For example, in the primary mass spectrum, peak **6** (Figure 3A) showed two prominent ion peaks at *m*/*z* 375 and 751. In the secondary mass spectrum, the intensity of the *m*/*z* 751 ion diminished, suggesting that *m*/*z* 375 represents the [M–H]^−^ ion peak and *m*/*z* 751 the [2M–H]^−^ ion peak. Additionally, the [2M+Na–2H]^−^ ion peak at *m*/*z* 773 in the primary mass spectrum confirmed the chemical formula C_16_H_24_O_10_ for this peak. In the secondary mass spectrum, constant shedding of CO_2_ (44 Da) and neutral glucose (162 Da) from the deprotonated molecule [M–H]^−^ of peak **6** generates fragment ions at *m*/*z* 213 and 169. The retro-Diels-Alder reaction involving *m*/*z* 169, followed by the loss of C_3_H_4_O (56 Da) and H_2_O (18 Da), resulted in distinctive fragment ions at *m*/*z* 113 and 151. Comparison with the standard confirmed that peak **6** is loganic acid. Figure 4A illustrates the proposed cleavage pathway.

#### 2.1.2. Phenylpropanoids

Phenylpropanoids, characterized by three straight-chain carbon linkages (C_6_-C_3_ group) and a benzene ring, are prevalent in *P. hookeri*. Most of these substances are caffeoylquinic acid derivatives, formed through the esterification condensation reaction of caffeic acid and quinic acid [18]. The distinctive quinic acid fragment ion *m*/*z* 191 frequently appears in the mass spectrum’s negative ion mode. For instance, in the primary mass spectrum, peak **7** (Figure 3B) exhibited two robust ion peaks at *m*/*z* 353 and 707. In the secondary mass spectrum, the intensity of the *m*/*z* 707 ion was reduced, indicating that *m*/*z* 353 corresponds to the [M–H]^−^ ion peak and *m*/*z* 707 to the [2M–H]^−^ ion peak. The [2M+Na–2H]^−^ ion peak at *m*/*z* 729 in the primary mass spectrum confirmed the chemical formula C_16_H_18_O_9_ for the peak. The fragment ions at *m*/*z* 191 and 179 in the secondary mass spectrum corresponded to the distinctive ions of quinic acid and caffeic acid, suggesting that it is a monocaffeoylquinic acid derivative. Comparison with the standard showed that peak **7** is chlorogenic acid. Peaks **3** and **10**, displaying the same *m*/*z* 353 quasi-molecular ion at different RTs, are isomers of peak **7**. Based on literature data and relative RTs, peaks **3** and **10** were identified as neochlorogenic acid and cryptochlorogenic acid, respectively. By using standard sample comparison and reviewing literature data, peaks **32**, **33**, and **36** were identified as di-caffeoylquinic acid derivatives, and relative RTs indicated that these peaks corresponded to isochlorogenic acid B, isochlorogenic acid A, and isochlorogenic acid C, respectively. Figure 4B illustrates the cleavage pathway of isochlorogenic acid C.

#### 2.1.3. Triterpenes

Pentacyclic triterpenoids, including ursolic and oleanolic acids, represent some of the primary active chemical constituents extensively found throughout *P. hookeri*. This class of molecules, through its core aglycone structure, attaches various monosaccharides at different substitution sites to form diverse triterpenoids. The varied monosaccharide connecting sequences in these compounds result in the production of numerous isomers. Given the stability of the aglycone structure in *P. hookeri*’s triterpenoids, the main changes observed in the mass spectrum arose from the loss of glycosyl groups such as glucosyl (Glc, 162 Da), rhamnosyl (Rha, 146 Da), and xylosyl (Xyl, 132 Da) [19]. In the primary mass spectrum, two ion peaks, *m*/*z* 1033 [M+Na–2H]^−^ and *m*/*z* 1011 [M–H]^−^, were detected with peak **87** (Figure 3C) as the subject of study. The chemical formula was determined to be C_52_H_84_O_19_. The secondary mass spectrum revealed ion peaks at *m*/*z* 865 [M–H–Rha]^−^, 733 [M–H–Rha–Xyl]^−^, 587 [M–H–2Rha–Xyl]^−^, and 455 [M–H–2Rha–2Xyl]^−^, illustrating the compound’s fragmentation pathway involving sequential loss of glycosyl groups. Based on a comparison with literature data, peak **87** was identified as triploside G. Figure 4C illustrates the cleavage pathway of triploside G.

### 2.2. FBMN Analysis

In recent years, metabolomics has become a widely used technique for analyzing quality indicators in Chinese herbal remedies. However, the classification model constructed may be distorted, and the *p*(corr) value could be affected by the substantial amount of orthogonal noise and irrelevant factors present in the original data. Additionally, employing a traditional VIP threshold of 1.0 leads to errors, such as false positives, due to an increase in the total number of input features used for model construction. By analyzing the similarity of secondary mass spectra among compounds in a sample, molecular networks allow these chemicals to form a discernible network structure. Grouping structurally related chemicals in the same network reduces error rates and diminishes the influence of human judgment. Increasing the response value threshold to filter data can reduce the likelihood of false positive errors.

**Figure 5:**
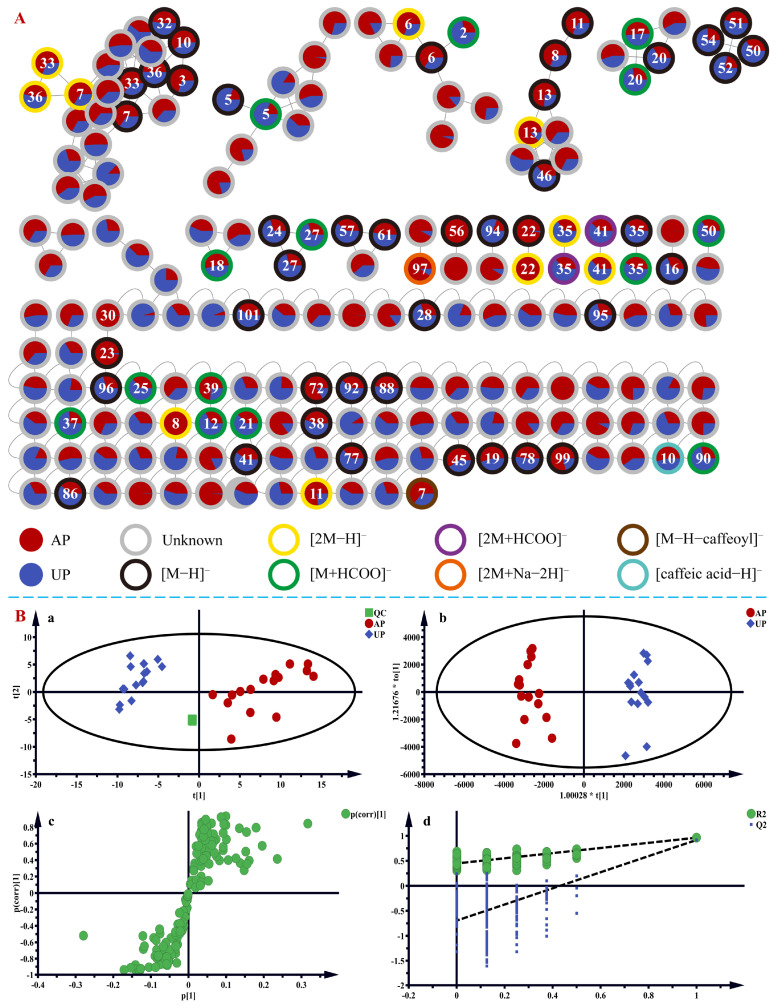
(**A**) The FBMN diagrams of the AP and UP of *P. hookeri* with response values greater than 10,000. (**B**) Multivariate statistical results of the AP and UP of *P. hookeri* with response values greater than 100,000. (**a**) PCA score plot; (**b**) OPLS-DA score plot; (**c**) OPLS-DA loadings plot; (**d**) Replacement test plot.

In Figure 5A, each node in the visual molecular network, representing *P. hookeri*’s aboveground and underground components, corresponds to a mass spectrum. Various colors of the nodes represent different components, with the extent of each color indicating the compound’s proportion in that component. The thickness of the edges connecting the nodes indicates the degree of similarity between their individual MS^2^ spectrum. The ring color around each node denotes different charged ion types. The connectivity of molecular clusters can further verify the accuracy of compound identification described above. The molecular network, constructed using the automatic extraction mode, contained 35,665 nodes (Figure S5). These nodes were further categorized on the basis of their differing response value levels. A total of 7868, 2771, 1669, 405, and 184 nodes had response values above 1000 (Figure S6), 5000 (Figure S7), 10,000 (Figure S8), 50,000 (Figure S9), and 100,000 (Figure 5A), respectively. Nodes exhibiting more than a 1.5-fold relative content difference (Relative Abundance Ratio > 1.5) were selected to identify potential chemical markers among various *P. hookeri* components. The efficacy of these chemical markers was confirmed through comparisons with the results of metabolomic analyses.

### 2.3. Metabolomics

**Figure 6:**
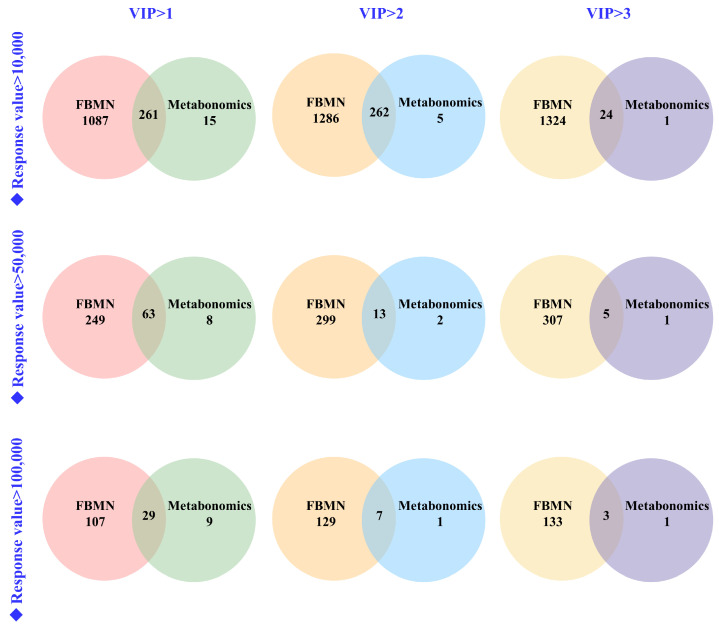
The comparison between metabolomics and FBMN conducted under various extraction procedures.

As shown in Figure 5B, PCA results displayed tight clustering of QC samples, confirming good stability of the established analytical method. Meanwhile, obvious separation was found between the AP and UP of *P. hookeri*. The OPLS-DA model further distinctly divided AP and UP samples into two independent groups, with corresponding response values exceeding 100,000. The results for the other response values are shown in Figures S10–S14. These results indicate a remarkable chemical composition difference between the AP and UP of *P. hookeri*. This study established thresholds with VIP values greater than 1, 2, and 3 as criteria for filtering out chemicals, thereby facilitating the identification of markers that distinguish different regions of *P. hookeri*. Metabolomics data, filtered using various VIP thresholds (VIP > 1, VIP > 2, and VIP > 3), were compared to nodes selected for FBMN, which had a relative content difference greater than 1.5 times. Figure 6 displays the comparison between metabolomics and FBMN conducted under various extraction procedures.

The data indicated that FBMN screens a substantially higher number of differential compounds than metabolomics under the same response conditions, with an overlap exceeding 80% between the results of FBMN and metabolomics. After a comprehensive comparison, FBMN findings with response values exceeding 100,000 were selected for further annotation analysis. Differential metabolites were characterized by having VIP values greater than 1 and response values exceeding 100,000. Following the removal of impurity peaks, in-source fragmentation peaks and adduct ion peaks, the key differential compounds tested included rivularicin (**86**), cantleyoside (**41**), sapindoside G-isomer (**79**), sylvestroside IV (**45**), hookeroside D-isomer (**84**), sapindoside G (**77**), isoorlentin (**22**), isoscoparin (**23**), swertisin (**30**), euscaphic acid (**72**), sylvestroside I (**35**), and isochlorogenic acid A (**33**). Table 2 demonstrates the VIP values of the 12 key difference compounds.

**Figure 7:**
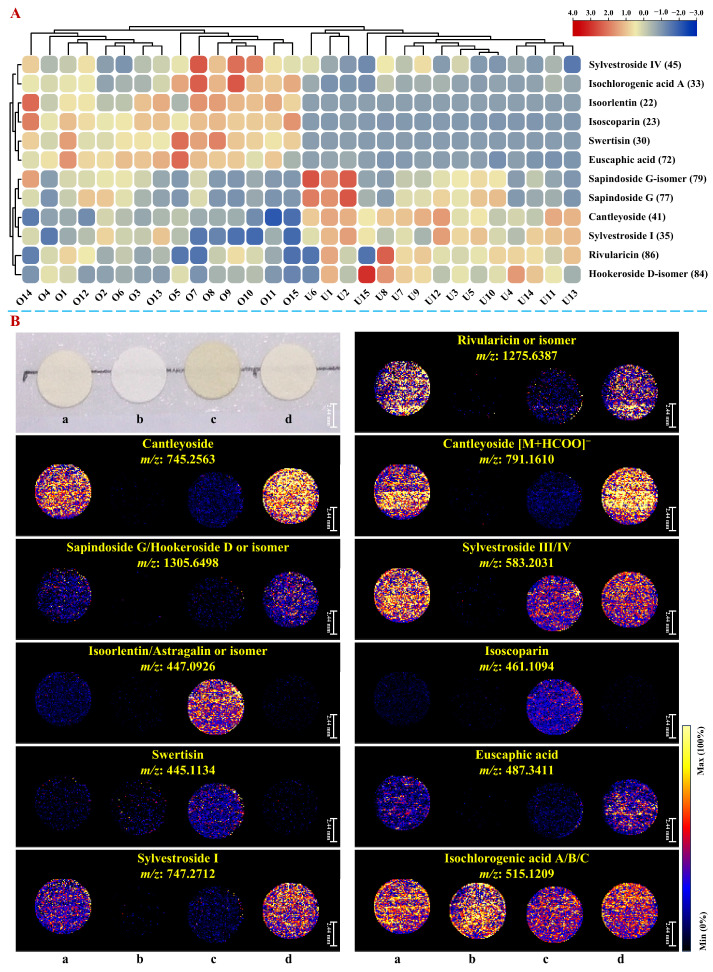
(**A**) The HCA heatmap of 12 differential compounds between AP and UP of *P. hookeri*. (**B**) PAD-DESI-MSI for different compounds in (**a**) Whole herb of *P. hookeri*, (**b**) mixed reference solution, (**c**) AP of *P. hookeri*, and (**d**) UP of *P. hookeri*.

To visualize the distribution of the contents of the 12 differential compounds in AP and UP of *P. hookeri*, HCA heatmap was generated with TBtools software (version 2.453). As shown in the heatmap (Figure 7A), rivularicin (**86**), cantleyoside (**41**), Sapindoside G-isomer (**79**), Hookeroside D-isomer (**84**), sapindoside G (**77**), and sylvestroside I (**35**) exhibited higher abundance in the UP of *P. hookeri*, whereas sylvestroside IV (**45**), isoorlentin (**22**), isoscoparin (**23**), swertisin (**30**), euscaphic acid (**72**), and isochlorogenic acid A (**33**) were predominantly localized in the AP of *P. hookeri*. 

### 2.4. PAD-DESI-MSI Characterisation of Different Makers in AP and UP of P. hookeri

The distribution of 12 differential components in AP and UP of *P. hookeri* was visualized using PAD-DESI-MSI. As shown in Figure 7B, rivularicin (**86**), cantleyoside (**41**), sapindoside G-isomer (**79**), hookeroside D-isomer (**84**), sapindoside G (**77**), and sylvestroside I (**35**) were predominantly localized in the UP of *P. hookeri*, whereas isoorlentin (**22**), isoscoparin (**23**), and swertisin (**30**) were primarily found in the AP, consistent with the heatmap analysis. Sylvestroside IV (**45**) and isochlorogenic acid A (**33**) could not be resolved in the imaging analysis owing to isomer interference in *P. hookeri*. In addition, the MSI result of the adduct ion [M+HCOO]^−^ of cantleyoside (**41**) show the same distribution as its quasimolecular ion [M−H]^−^.

The twelve screened differential compounds comprised three iridoids, three flavonoids, five triterpenes, and one phenylpropanoid. Among these metabolites, flavonoids and triterpenoids displayed high ion abundance in mass spectra, yet they have seldom been quantitatively determined in previous studies. Regarding iridoids, cantleyoside and sylvestroside I have been repeatedly quantified in prior investigations. In comparison, sylvestroside IV is difficult to accurately quantify owing to its chromatographic co-elution with its isomer sylvestroside III. In general, the three iridoids are abundantly distributed in *P. hookeri*, with cantleyoside present at the highest concentration [20]. Pharmacological research has validated that these differential metabolites exert anti-inflammatory, antioxidant, and analgesic activities [21]. Specifically, cantleyoside has been proven to ameliorate inflammatory responses in rheumatoid arthritis, which is highly consistent with the traditional medicinal properties of *P. hookeri* [22]. The majority of these discriminatory metabolites serve not only as diagnostic chemical markers for distinguishing plant tissues but also as core bioactive substances responsible for the herb’s medicinal efficacy. Collectively, the twelve differential metabolites clearly characterize the chemical divergence between AP and UP of *P. hookeri*, acting as reliable chemical fingerprints for botanical identification and crucial bioactive contributors to its therapeutic value.

## 3. Discussion

Untargeted metabolomics employs three primary modes of data acquisition: full scan, data-dependent acquisition (DDA), and data-independent acquisition (DIA). In full scan mode, the method captures information on all ions in the sample, allowing for the inference of molecular formulas from accurate masses and isotopic patterns of ions. This mode limits detailed structural identification because it does not involve ion fragmentation, thereby precluding MS/MS (MS^2^) analysis for collecting fragment ion information [23,24]. Consequently, researchers frequently use full scan mode alongside other acquisition modes for investigating smaller systems. In DDA mode, the MS^2^ scan specifically targets ions detected in the full scan (MS^1^). Following each MS^1^ scan, several ions, typically based on their intensity, are selected and sequentially fragmented in MS^2^ scans [25]. DIA acquires MS^2^ spectra of all ions without needing predefined criteria for ion fragmentation. DIA modes may incorporate selected ion monitoring, where chosen ions fragment within a specified mass window, or all-ion fragmentation, where fragmentation is applied to all ions [26]. The spectral quality in DDA is superior to that in DIA, more closely resembling the reference spectrum. However, DIA can collect more MS^2^ spectra than DDA. Conversely, DDA offers easier access to higher-quality spectra but often struggles with complex chemical compositions [27].

Studies have shown that DIA obviously outperforms DDA in the qualitative analysis of complex samples, including whole cell and traditional Chinese medicine samples [28]. However, MS^2^ data analyzed using DIA methods may produce false positives [29]. Thus, the present study employed the MS^E^ mode (analogous to DIA) of the Waters Company UPLC-Q-TOF/MS to collect mass spectra from various *P. hookeri* components, effectively reducing false positives by increasing the response value threshold. Research indicated that integrating DIA and DDA techniques offers complementary insights for metabolite identification [30]. The team plans to gather data by using the DDA mode in future studies and compare it with existing results to enhance the research outcomes.

In this study, commonly employed multivariate statistical analysis methods in metabolomics were applied to screen differential compounds in various parts of *P. hookeri*. The results were compared with those obtained from FBMN. Among all detected response profiles, FBMN annotated far more differential compounds than OPLS-DA. OPLS-DA is a supervised discriminant algorithm that filters variations in the independent variable X irrelevant to the categorical variable Y. It mainly concentrates classification-related information into one principal component, removes redundant noise to simplify the model, and further improves the analytical performance and reliability [31]. In contrast, FBMN characterizes the relative distribution of ion peak intensities across different samples and clusters ions according to metadata grouping. Its results are affected by chromatographic detection and data processing workflows rather than dimensionality reduction procedures, enabling FBMN to capture more differentially distributed compounds [32]. Therefore, OPLS-DA exhibits prominent superiority in eliminating interfering variables and generating concise and interpretable results. By contrast, FBMN is capable of capturing variations in low-abundance compounds by appropriately lowering the response threshold during chromatographic data processing. This unique feature endows FBMN with great value for exploring subtle metabolic alterations in drug metabolism and in vivo microbial metabolic studies [33].

Molecular networking has emerged as a pivotal technology for visualizing and annotating chemical structures in untargeted MS data. During the chromatographic elution in untargeted LC-MS/MS data acquisition, the same precursor ion can fragment multiple times. In CLMN using MS-Cluster, spectra are typically clustered into a single node, though occasionally multiple nodes are generated to represent the same chemical [34]. Conversely, FBMN integrates relative quantitation information with other key mass spectrometric data (such as precursor isotope patterns and adduct annotations) to streamline and synthesize the data, whereas CLMN simply tallies the spectra or total precursor ions. This approach reduces the likelihood of identifying compounds with similar structures [35]. Additionally, FBMN facilitates effective visualization and annotation of isomers in LC-MS/MS data by combining relative quantitation with ion flux data.

According to the study, FBMN exhibits numerous disconnected nodes, potentially limiting the distribution of metabolites annotated by the database within the network and causing unnecessary fragmentation of molecular families (subnetworks). Ion identity molecular networking (IIMN) can address issues related to excessive feature redundancy in MS metabolomics and the presence of disconnected adduct ions in classical molecular networks. IIMN involves peak identification, peak grouping, and ion identification on the basis of feature shape correlation to link structurally similar compounds via MS^2^ similarity and various adduct ions of the same chemical by using MS^1^ features. By integrating this network with the use of two layers of features, the molecular network increases its annotation density, reduces the number of disconnected nodes, and decreases the redundancy of distinct adducted ion forms of the same molecule [36].

Although FBMN can distinguish isomers, it struggles to identify novel molecules with fundamentally different structures. A viable alternative is block-based molecular networking (BBMN), which offers several advantages: (1) High selectivity: It identifies compounds related to biological genes by detecting characteristic fragments from specific organisms’ gene structures; (2) Speed: It optimizes extensive MS^2^ data into a more streamlined and focused data set, accelerating further data analysis. This method is effective for discovering new plant-based natural products and can be applied to investigate natural products in animals, microbes, and marine organisms [37].

Despite MSI in spatial distribution analysis of plant natural products, its application remains technically challenging for isomer differentiation [38]. The results demonstrate that isochlorogenic acid A (**33**) exhibited specific distribution in AP of *P. hookeri* according to heatmap analysis, whereas the distribution of *m*/*z* 515 in MSI showed no significant variation. This is because its distribution pattern is opposite to that of its isomer isochlorogenic acid B (**32**), isochlorogenic acid C (**36)**, but its abundance is comparable. A parallel observation was made for sylvestroside IV (**45**). In contrast, sapindoside G-isomer (**79**) and hookeroside D-isomer (**84**), along with sapindoside G (**77**), despite sharing identical *m*/*z* 1305, displayed distinct spatial distributions in MSI due to their consistent localization patterns. Notably, euscaphic acid (**72**) manifested a distribution profile in MSI that was diametrically opposed to the heatmap, strongly indicating coexisting isomers with divergent distribution trends and markedly different concentrations. Collectively, these findings highlight the necessity of validating MSI data with complementary mass spectrometry techniques. Beyond mass spectrometry-based platforms, other conventional analytical approaches can also serve as reliable auxiliary tools to consolidate MSI results. For instance, ultraviolet-visible spectroscopy and high-performance liquid chromatography enable quantitative verification of characteristic chemical constituents and eliminate spectral interference caused by complex plant matrices. Additionally, chemometric methods, including hierarchical clustering and correlation analysis, can further confirm the consistency between spatial distribution patterns and relative compound abundance. The combination of multiple independent analytical strategies minimizes instrumental bias and matrix interference, thereby improving the reproducibility and methodological robustness of MSI visualization results. Therefore, cross-validation incorporating both spectrometric and non-spectrometric techniques is strongly recommended for future phytochemical spatial investigations of medicinal plants.

## 4. Materials and Methods

### 4.1. Materials and Reagents

The reference standards of oleanolic acid, ursolic acid, and chlorogenic acid were obtained from the China National Institute for Food and Drug Control in Beijing, China. Loganin, isochlorogenic acid C, dipsanoside B, and sylvestroside Ι were sourced from Shanghai Yuaye Bio-Technology Co., Ltd. (Shanghai, China). Cantleyoside was acquired from Sichuan PurChemland Standard Technology Co., Ltd. (Chengdu, China). Sweroside and dipsanoside A were previously isolated from *P. hookeri* in earlier studies. Methanol (MeOH) and acetonitrile (ACN) of LC/MS grade were obtained from Avantor (Radnor, PA, USA), and leucine enkephalin (LE) and formic acid (FA) were sourced from Sigma-Aldrich (St. Louis, MO, USA). Distilled water was supplied by Watsons Food and Beverage Co., Ltd. (Guangzhou, China), and all other reagents were of analytical grade.

Fifteen batches of *P. hookeri* samples, comprising AP and UP, were collected from Yunnan and Sichuan Provinces. All herbs were authenticated by Associate Professor Ce Tang of Chengdu University of TCM in accordance with the ChP 2025 and subsequently deposited at the School of Ethnic Medicine, Chengdu University of TCM.

### 4.2. Sample Preparation

Thirty batches of samples were powdered and sieved using a 355 μm mesh (Chinese National Standard Sieve No. 3). Subsequently, 2.0 g of the sieved material was transferred into a 100 mL conical flask with a stopper. Then, 50 mL of 70% aqueous MeOH (*v*/*v*) was added. The flask was weighed before being subjected to ultrasonic extraction in a 30 °C water bath (250 W, 40 kHz) for 30 min. After being cooled to room temperature, the evaporated volume was replenished with 70% aqueous MeOH to the original weight, and the solution was then filtered. The residue was rinsed with a small volume of 70% aqueous MeOH. Afterwards, the collected filtrates were concentrated and redissolved in the same solvent. The concentrated extract was transferred into a 10 mL volumetric flask, filled up to the mark with 70% MeOH. This solution was then centrifuged at 13,000 rpm for 15 min and passed through a 0.22 μm microporous membrane. Then, 100 μL from each sample was combined to create quality control (QC) samples to maintain the consistency and stability of MS analyses. QC injections were randomly conducted throughout the sample collection process.

### 4.3. UPLC-Q-TOF/MS Analysis

UPLC analysis was conducted on a Waters ACQUITY UPLC system, which includes a binary solvent delivery manager, an auto-sampler, and the Empower Chromatographic Workstation (Waters Corporation, Milford, MA, USA). Separation was achieved using an ACQUITY UPLC BEH C18 (2.1 mm × 100 mm, 1.7 μm) column. The sample and QC injection volumes were standardized at 2 μL. Flow rate and column temperature were maintained at 0.4 mL/min and 40 °C, respectively. The mobile phase was composed of 0.1% FA in water (A) and 0.1% FA in ACN (B). The gradient program was administered as follows: 0–7 min, 95–88% B; 7–8 min, 88–85% B; 8–20 min, 85–83% B; 20–30 min, 83–75% B; 30–33 min, 75–60% B; 33–48 min, 60% B steady; 48–55 min, 60–40% B; 55–57 min, 40–15% B; 57–60 min, 15–1% B.

MS analyses were carried out with a Waters Q-TOF analyzer configured within the SYNAPT G2 HDMS system (Waters Corporation, Milford, MA, USA), which had an electrospray ionization source. The analysis conditions were set as follows: atomization and cone hole gas utilized nitrogen; the source temperature was 150 °C; cone gas flow was established at 50 L/h; desolvation was conducted at 450 °C with a gas flow rate of 800 L/h; the voltages for the sampling and extraction cones were set at 40 and 4 V, respectively; the capillary voltage varied, being 3.0 kV in positive mode and 2.5 kV in negative mode; the scan and interscan times were 0.3 and 0.02 s, respectively; and the mass-to-charge ratio ranged from *m*/*z* 100 to 1700 Da. Lock mass adjustments were performed using the LE internal standard. Lock masses were measured in positive ion mode *m*/*z* 556.2771 [M+H]^+^ and in negative ion mode *m*/*z* 554.2615 [M–H]^−^. The system and data acquisition were controlled using MassLynx software (version 4.2, Waters Corporation, Milford, MA, USA).

### 4.4. Data Collection and Processing

Data acquisition was conducted using MassLynx software, followed by importing of the data into Progenesis QI for tasks such as peak identification, alignment, baseline correction, deconvolution, and normalization. This processing generated a quantization table (CSV format) and an MS/MS spectral summary file (MSP format). Data files were extracted on the basis of response values exceeding the thresholds of 1000, 5000, 10,000, 50,000, and 100,000, facilitating the aggregation of varied response data. The corresponding CSV, MSP files, and metadata tables were uploaded to the GNPS (http://gnps.ucsd.edu) (accessed on 14 May 2024) platform. FBMN files were generated. Within the analysis and calculation interface, the data source was set to Progenesis QI, with the ion mass error tolerance for precursor and fragment ions established at 0.02 Da. The advanced network settings were adjusted as follows: minimum pairs cosine value to 0.7, minimum matched fragment ions to 6, network TopK to 10, and maximum connected component size (beta) to 100. Within the advanced multivariate statistics settings, the PCoA distance metric was configured to cosine to establish molecular networks. The results of the FBMN analysis could be accessed at the following link: https://gnps.ucsd.edu/ProteoSAFe/status.jsp?task=8d51f61c58a6452ebf640a4ccc5037e4 (accessed on 14 May 2024), https://gnps.ucsd.edu/ProteoSAFe/status.jsp?task=4d873da880f84af6ad18af1b16a1b9eb (accessed on 14 May 2024), https://gnps.ucsd.edu/ProteoSAFe/status.jsp?task=2ed14b05040146b2883949befa1e0d54 (accessed on 14 May 2024), https://gnps.ucsd.edu/ProteoSAFe/status.jsp?task=1d1b3eda3ad1439b8df31cbafb88fe40 (accessed on 14 May 2024), https://gnps.ucsd.edu/ProteoSAFe/status.jsp?task=6ad30123a4d548aaa4704064377cd71d (accessed on 14 May 2024). The correlation network was imported into Cytoscape (version 3.10.1) for visualization and network analysis.

### 4.5. Multivariate Statistical Analysis

Raw files underwent processing in Progenesis QI software (version 2.4), which involved denoising, peak identification, alignment, and normalization. Subsequently, a data matrix with RT, mass-to-charge ratio (*m*/*z*) values, and peak intensity was automatically generated. Matrices containing response values exceeding the thresholds of 1000, 5000, 10,000, 50,000, and 100,000 were further extracted. The extracted data matrix files were subsequently imported into SIMCA (version 14.1, Umetrics, Umea, Sweden) for multidimensional statistical analysis, which included conducting unsupervised principal component analysis (PCA) and supervised orthogonal partial least square-discriminant analysis (OPLS-DA). An S-plot was employed to visualize the predictive component loadings from OPLS-DA to aid in identifying chemical markers. The markers were screened using VIP scores, and metabolites with a VIP value exceeding 1 were regarded as potential discriminative markers.

### 4.6. PAD Analysis

As a facile, rapid, cost-effective and environment-friendly material, qualitative filter paper was used for PAD fabrication. The filter paper was first cut into circles in 1 cm diameter. The extract solution for whole herb, AP and UP of *P. hookeri*, and the mixed standard solution with a volume of 1 μL were continuously sampled onto the filter papers by using a pipette gun. The paper was then dried at room temperature and pasted in the glass slide for DESI-MSI analysis.

### 4.7. DESI-MSI Detection

Imaging experiments were conducted using a Waters SYNAPT QTof mass spectrometer equipped with a DESI source (Waters Corporation, Milford, MA, USA). The following DESI parameters were optimized to obtain good signal intensity: nebulizing gas (dry nitrogen): 0.45 MPa, spray solvent: 70% MeOH water, 30% H_2_O, 0.1% FA, and 0.1 mM LE at a flow rate of 2 μL/min, capillary voltage: 3.0 kV, and ionization mode: negative. The pixel size (150 μm X and Y pixel size) was determined based on the total scanning time of the mass spectrogram and the speed of the X–Y pixel size scanner. The mass range was *m*/*z* 100–1000, and the scanning speeds was 100 μm/s for PAD.

HDI software (version 1.5) was used to process raw MS files as well as create and view MS images. The MS raw data file was imported into HDI for imaging, and the regions of interests (ROIs) expanded by four points were exported.

## 5. Conclusions

In summary, this study identified 101 chemicals and further clarified the chemical basis for the therapeutic applicability of *P. hookeri*, using UPLC-Q-TOF/MS to extensively analyze its chemical components. Twelve differential chemical compounds were analyzed alongside various sections of *P. hookeri* by using metabolomics integrated with FBMN. The distribution of these 12 compounds in AP and UP of *P. hookeri* was also visually characterized by HCA heatmap and PAD-DESI-MSI. This study introduced new concepts for utilizing molecular networking and not only provided a theoretical basis for the quality control of *P. hookeri* but also offered insights into optimizing the medicinal site.

## Figures and Tables

**Table 1:** MS data for compounds identified from *P. hookeri* in negative ion mode.

No.	R_t_ (min)	Identification	Formula	MS /[M−H]^−^	δ (ppm)	Fragment Ions	Types
1	1.58	Unknown 1	C_19_H_20_O_10_	407.1553	1.5	815.2354, 453.1608, 429.0099	Unknown
2	2.11	Ptehoside B	C_15_H_24_O_8_	331.1388	−1.5	663.2866, 377.1449, 185.0811, 169.0864	Iridoid
3	2.24	Neochlorogenic acid	C_16_H_18_O_9_	353.0872	−0.3	191.0552, 179.0344, 173.0815, 161.0458, 135.0444	Phenylpropanoid
4	2.47	Phyllaemblicin D	C_21_H_34_O_13_	493.1920	−0.2	987.3807, 539.1970, 331.1398	Others
5	2.60	Unknown 2	C_20_H_32_O_12_	463.1813	−0.6	927.3705, 509.1875, 331.1388	Unknown
6	3.24	Loganic acid	C_16_H_24_O_10_	375.1309	4.8	773.2487, 751.2690, 213.0757, 169.0865, 151.0757, 113.0238	Iridoid
7	3.60	Chlorogenic acid *	C_16_H_18_O_9_	353.0877	1.1	729.1646, 707.1831, 375.0692, 191.0555, 179.0348, 173.0455, 161.0240, 135.0446	Phenylpropanoid
8	3.95	8-Epiloganic acid	C_16_H_24_O_10_	375.1292	0.3	751.2661, 397.1107, 213.0762, 169.0865, 151.0759	Iridoid
9	4.06	Caffeic acid	C_9_H_8_O_4_	179.0347	1.7	201.1125, 135.0448	Phenylpropanoid
10	4.11	Cryptochlorogenic acid	C_16_H_18_O_9_	353.0878	1.4	375.0695, 191.0555, 179.0346, 173.0451, 161.0235, 135.0448	Phenylpropanoid
11	4.31	Deacetylasperulosidic acid	C_16_H_22_O_11_	389.1083	−0.3	779.2249, 345.1186, 183.0657	Iridoid
12	4.41	Unknown 3	C_32_H_22_O_4_	469.1452	2.6	939.2987, 537.1823	Unknown
13	4.69	Swertimarin	C_16_H_22_O_10_	373.1133	−0.5	769.2165, 747.2346, 211.0238, 193.0496, 167.0709, 149.0600	Iridoid
14	4.89	3′-*O*-β-D-glucopyranosyl sweroside	C_22_H_32_O_14_	519.1717	0.6	1039.3558, 357.1309, 195.0655, 125.0237	Iridoid
15	5.57	7-Epiloganin	C_17_H_26_O_10_	389.1448	0.0	227.0920, 209.0825	Iridoid
16	5.76	Berchemol 4′-*O*-β-D-glucoside	C_26_H_34_O_12_	537.1971	−0.2	1075.3994, 583.2037, 375.1443	Phenylpropanoid
17	6.06	Sweroside *	C_16_H_22_O_9_	357.1181	−1.4	715.2436, 403.1268, 125.0235	Iridoid
18	6.20	Loganin *	C_17_H_26_O_10_	413.1422 /[M+Na]^+^	−0.5	435.1501	Iridoid
19	6.59	Dipsanoside H	C_22_H_32_O_14_	519.1720	1.2	1039.3514, 565.1772, 357.1238, 195.0656, 125.0237	Iridoid
20	6.78	6′-*O*-beta-Apiofuranosylsweroside	C_21_H_30_O_13_	489.1613	1.0	979.3301, 535.1670, 357.1511, 339.0517, 195.0655, 149.0453, 125.0241	Iridoid
21	7.09	(7R,8S)-erythro-7,9,9′-trihydroxy-3,3′-dimethoxy-8-*O*-4′-neolignan-4-*O*-β-D-glucoside	C_26_H_36_O_12_	539.2133	0.7	585.2183, 377.1603	Iridoid
22	7.69	Isoorlentin	C_21_H_20_O_11_	447.0926	−0.2	895.1947, 285.0398	Others
23	8.29	Isoscoparin	C_22_H_22_O_11_	461.1094	2.2	923.2253, 299.0514	Others
24	8.92	8-Hydroxypinoresinol-4′-*O*-β-D-glucopyranoside	C_26_H_32_O_12_	535.1816	0.0	581.1862, 557.1829, 373.1281, 343.1182, 313.1072	Iridoid
25	9.04	(-)-Syringaresinol diglucoside	C_34_H_46_O_18_	741.2637	0.1	787.2659, 579.2072, 417.1552	Phenylpropanoid
26	9.10	Apigenin 7-*O*-glucoside	C_21_H_20_O_10_	431.0973	−1.2	477.1958, 269.0452	Others
27	9.16	8′-Hydroxylpinoresionl-4-*O*-β-D-glucoside	C_26_H_32_O_12_	535.1818	0.4	581.1868, 373.1281, 343.1182, 313.1071	Iridoid
28	9.54	Caryocanoside B	C_32_H_46_O_19_	733.2548	−1.0	1467.5178, 571.2029, 409.1495	Iridoid
29	9.61	Astragalin	C_21_H_20_O_11_	447.0928	0.2	285.0392, 284.0324, 151.0031	Others
30	9.68	Swertisin	C_22_H_22_O_10_	445.1134	−0.2		Others
31	10.63	Unknown 4	C_26_H_28_O_14_	563.1398	−0.5	1127.3192, 609.1440, 431.1053, 269.0451	Others
32	10.84	Isochlorogenic acid B	C_25_H_24_O_12_	515.1190	0.0	1053.2260, 537.1002, 353.0867, 191.0549, 179.0561, 173.0451, 161.0240	Phenylpropanoid
33	11.04	Isochlorogenic acid A	C_25_H_24_O_12_	515.1209	3.7	1053.2273, 1031.2460, 537.1007, 353.0877, 191.0555, 179.0343, 173.0452, 135.0446	Phenylpropanoid
34	12.25	Foliasalacioside B_1_	C_24_H_40_O_11_	503.2509	3.4	1007.3760, 549.2554, 371.2073, 239.0920	Others
35	12.84	Sylvestroside I *	C_33_H_48_O_19_	747.2712	0.0	1541.5579, 1495.5508^−^, 793.2795, 585.2172, 567.2087	Iridoid
36	13.41	Isochlorogenic acid C *	C_25_H_24_O_12_	515.1215	4.9	1053.2263, 1031.2462, 537.1002, 353.0877, 191.0554, 179.0344, 173.0452, 135.0447	Phenylpropanoid
37	13.71	Unknown 5	C_25_H_36_O_12_	527.2136	1.3	1055.4354, 573.2191, 365.1604	Others
38	14.16	Loganin pentaacetate	C_27_H_36_O_15_	599.1979	0.5	1199.4147	Iridoid
39	14.76	Laciniatoside I-isomer	C_27_H_38_O_14_	585.2181	−0.3	631.2234	Iridoid
40	15.02	Laciniatoside I-isomer	C_27_H_38_O_14_	585.2182	−0.2	631.2250, 423.1652	Iridoid
41	16.11	Cantleyoside *	C_33_H_46_O_19_	745.2563	1.1	1537.5278, 1491.5200, 791.1610, 583.2024	Iridoid
42	17.75	Laciniatoside I	C_27_H_38_O_14_	585.2175	1.4	631.2234, 423.1623, 405.1562, 195.0653	Iridoid
43	18.24	Strychoside B	C_66_H_90_O_37_	1473.5085	0.1	1519.5153, 1311.4575, 1293.4463, 1131.4398, 969.3819, 825.2817	Iridoid
44	21.41	Sylvestroside III	C_27_H_36_O_14_	583.2031	0.7	1189.3962, 421.1496, 373.1135, 211.0603, 193.0503	Iridoid
45	21.54	Sylvestroside IV	C_27_H_36_O_14_	583.2037	1.7	1189.3962, 421.1505, 373.1137, 211.0604, 193.0500	Iridoid
46	22.27	Pterocephaline	C_44_H_56_N_2_O_20_	931.3361	−1.8		Others
47	22.84	Unknown 6	C_53_H_72_O_30_	1187.4044	1.2	1233.4099, 1025.3521	Triterpene
48	24.15	Triplostoside A	C_35_H_52_O_20_	791.2966	−1.0	837.3027, 629.2369, 495.1496, 467.1554	Iridoid
49	25.35	Pterocenoid C	C_22_H_30_O_10_	453.1759	−0.4	907.2989	Iridoid
50	25.95	Dipsanoside B	C_66_H_90_O_37_	1473.5093	0.7	1519.5151, 1311.4576, 1293.4482, 1131.4398, 969.3478, 825.3179	Iridoid
51	26.39	Dipsanoside A	C_66_H_90_O_37_	1473.5087	0.3	1311.4558, 1293.4452, 1149.4009, 1131.3817, 969.3433, 807.2203, 789.1618	Iridoid
52	27.25	Pterocephanoside A	C_58_H_78_O_30_	1253.4529	2.9	1299.4594, 1091.4491	Triterpene
53	28.35	Sylvestroside IV dimethyl acetal	C_29_H_42_O_15_	629.2449	0.6	1259.3995, 651.2348	Iridoid
54	28.68	Unknown 7	C_58_H_78_O_30_	1253.4509	0.7	1299.4575	Triterpene
55	29.28	Unknown 8	C_50_H_66_O_23_	1033.3916	−0.1	1079.3986, 871.3392, 691.2740	Unknown
56	31.30	Kaempferol-3-*O*-(3″,6″-di-*O*-E-p-coumaroyl)-β-D-glucopyranoside	C_32_H_28_O_14_	635.1404	0.5	1271.2900	Others
57	32.76	Semipapposide D	C_75_H_122_O_38_	1629.7537	0.1	1675.7596, 1483.6965, 1305.6498, 1159.5908, 1027.5475, 865.4911, 733.4515, 583.2020, 455.3505	Triterpene
58	32.84	Hookeroside C	C_75_H_122_O_38_	1629.7548	0.7	1675.7601, 1483.6958, 1305.6499, 1159.5908, 1027.5488, 865.4955, 733.4527, 583.3194, 455.3524	Triterpene
59	32.87	Hookeroside B	C_69_H_112_O_34_	1483.6960	0.2	1529.7028, 1351.6548, 159.5907, 1027.5494, 865.3359, 733.2751, 583.9886, 455.9868	Triterpene
60	32.91	Hookeroside A	C_64_H_104_O_30_	1351.6548	1.0	1027.5470, 865.4976, 733.2747, 583.2219, 455.1821	Triterpene
61	33.13	Unknown 9	C_75_H_122_O_38_	1629.7556	1.2	1675.7615, 1497.7114, 1305.6497, 1173.6058, 1027.5471, 1011.5523, 895.5052, 733.4522, 583.2024, 455.3519	Triterpene
62	33.26	Scoposide G	C_58_H_94_O_25_	1189.6018	1.6	1235.6045, 1027.5480, 865.4966, 733.4531, 583.3954, 455.3498	Triterpene
63	33.40	3-*O*-α-L-Rha-(1→2)-α-L-Ara Hederagenin-28-*O*-β-D-xylyl-(1→6)-β-D-Glc ester	C_53_H_86_O_21_	1057.5599	1.5	1103.5616, 911.4805, 733.4540	Triterpene
64	33.99	Songoroside M-isomer	C_63_H_102_O_29_	1321.6450	1.6	1367.6504, 1189.5863, 1159.5923, 1057.5154, 1027.5485, 911.4771	Triterpene
65	34.38	*O*-β-D-Glc-(1→2)-*O*-[β-D-Xyl-(1→3)]-*O*-β-D-Xyl-(1→2)-β-D-Glc (3β)-3-[(2-*O*-β-D-Xyl-β-D-Glc)oxy]olean-12-en-28-oate	C_63_H_102_O_30_	1337.6399	1.6	1383.6449, 1205.5952, 1043.5397, 881.4915, 749.4473, 617.3654, 455.3169	Triterpene
66	34.63	Yemuoside YM_21_	C_63_H_100_O_29_	1319.6299	2.0	1173.5837, 1011.5547, 865.4972, 733.4529, 703.4442, 571.1891, 439.1516	Triterpene
67	34.63	Dipsacus saponin P	C_58_H_94_O_26_	1205.5972	1.4	1251.6046, 1073.6396, 1043.5421, 911.4104, 881.4858, 749.4489, 603.3723, 471.3124	Triterpene
68	34.76	Scoposide E	C_46_H_74_O_15_	865.4958	1.0	733.4525, 587.3941, 455.2517	Triterpene
69	34.84	Virgaureasaponin B	C_71_H_114_O_33_	1493.7185	1.4	1539.7247	Triterpene
70	35.10	Eupteleasaponin III	C_63_H_100_O_29_	1319.6299	2.0	1187.5848, 1025.5313, 879.4474, 733.4525, 571.1901, 439.1534	Triterpene
71	35.17	Eupteleasaponin II	C_58_H_92_O_25_	1187.5862	1.1	1041.5277, 1025.5317, 879.4883, 733.4531, 571.1880, 439.1541	Triterpene
72	36.79	Euscaphic acid	C_30_H_48_O_5_	487.3411	1.6	533.3489	Triterpene
73	38.64	Scabioside F	C_57_H_92_O_24_	1159.5903	0.3	1027.5476, 881.4885, 749.4464, 587.1869, 455.3508	Triterpene
74	39.00	Songoroside M	C_63_H_102_O_29_	1321.6445	1.2	1159.5896, 1027.5475, 865.2826, 733.2599, 587.3919, 455.3541	Triterpene
75	39.50	Pterohoonoid C	C_30_H_44_O_5_	483.3112	0.4		Triterpene
76	40.45	Pterohoonoid A	C_30_H_44_O_5_	483.3120	2.1		Triterpene
77	41.05	Sapindoside G	C_63_H_102_O_28_	1305.6498	1.5	1173.6028, 1159.5898, 1027.5464, 865.4963, 733.4505, 601.3730, 455.3544	Triterpene
78	41.50	Prosapogenin Bx	C_57_H_92_O_24_	1159.5909	0.8	1027.5481, 881.4912, 749.4444, 587.3951, 455.3168	Triterpene
79	42.19	Sapindoside G-isomer	C_63_H_102_O_28_	1305.6519	3.1	1173.6058,1159.5898,1027.5483, 865.4969, 733.4496, 587.3946, 455.3531	Triterpene
80	42.68	3-*O*-β-D-Xyl-(1 →4)-β-D-Glc-(1→4)-β-D-Xyl-(1→3)-α-L-Rha-(1→2)-α-L-Xyl oleanolic acid	C_57_H_92_O_24_	1159.5917	1.5	1027.5485, 865.4977, 733.4524, 587.3942, 455.3537	Triterpene
81	43.18	Prosapogenin Ax	C_52_H_84_O_20_	1027.5483	0.5	865.4947, 733.4529, 587.3946, 455.3522	Triterpene
82	43.18	Bretschnoside A	C_64_H_104_O_29_	1335.6599	1.0		Triterpene
83	44.05	Hookeroside D	C_63_H_102_O_28_	1305.6519	3.1	1173.6066, 1159.5914, 1027.5515, 997.5390, 865.4995, 733.4496, 587.3946, 455.3539	Triterpene
84	46.17	Hookeroside D-isomer	C_63_H_102_O_28_	1305.6486	0.5	1173.6046, 1027.5443, 881.4911, 749.4470, 587.3942, 455.3520	Triterpene
85	46.56	Pterocephin A	C_62_H_100_O_27_	1275.6390	1.3	1297.6219, 1143.5967, 1011.5542, 865.4957, 733.4534, 587.3942, 455.3529	Triterpene
86	47.37	Rivularicin	C_62_H_100_O_27_	1275.6387	1.0	1143.5956, 1011.5529, 997.5388, 865.4949, 733.4513, 587.3947, 455.3167	Triterpene
87	48.53	Triploside G	C_52_H_84_O_19_	1011.5540	1.1	1033.5309, 865.4958, 733.4520, 587.3942, 455.3518	Triterpene
88	48.93	Prosapogenins Ax-3	C_46_H_74_O_15_	865.4960	1.3	1731.9994, 733.4518, 587.3938, 455.3525	Triterpene
89	51.30	Pterohoonoid D	C_30_H_46_O_4_	469.3322	0.9	939.6722	Triterpene
90	51.42	Giganteaside D	C_41_H_66_O_11_	733.4524	−0.4	1467.9137, 779.4584, 587.3947, 455.3528	Triterpene
91	52.29	11-Oxooleanolic acid	C_30_H_46_O_4_	469.3318	0.0	939.6722	Triterpene
92	53.00	Songoroside A-isomer	C_35_H_56_O_7_	587.3945	−0.5	633.4013, 455.9790	Triterpene
93	53.12	Schekwangsienin	C_30_H_44_O_4_	467.3174	2.8		Triterpene
94	53.27	Pterohoonoid B	C_30_H_42_O_4_	465.3006	0.2	931.6088	Triterpene
95	53.89	Songoroside A	C_35_H_56_O_7_	587.3944	−0.7	1175.7971, 633.4009	Triterpene
96	54.56	Atricin A	C_30_H_46_O_4_	469.3328	2.1	939.6716	Triterpene
97	55.06	Schekwangsienin-isomer	C_30_H_44_O_4_	467.3169	1.7	935.6406	Triterpene
98	56.66	Hookeroside D-isomer	C_63_H_102_O_28_	1305.6503	1.8		Triterpene
99	57.62	Oleanolic acid	C_30_H_48_O_3_	455.3532	1.5	933.6559, 911.7142	Triterpene
100	57.67	Ursolic acid *	C_30_H_48_O_3_	455.3533	1.8	933.6567, 911.7139	Triterpene
101	58.24	Oleanonic acid *	C_30_H_46_O_3_	453.3386	3.7	907.6822	Triterpene

* Identified with standards. Glc = glucopyranosyl, Rha = rhamnopyranosyl, Xyl = xylopyranosyl, Ara = arabinopyranosyl.

**Table 2:** The VIP values of 12 key differential compounds.

*m*/*z*	Retention Time (min)	VIP	Compound
1275.6385	47.37	3.70	Rivularicin (**86**)
745.2556	16.14	3.35	Cantleyoside (**41**)
1305.6488	42.19	3.26	Sapindoside G-isomer (**79**)
583.2035	21.54	3.13	Sylvestroside IV (**45**)
1305.6492	46.17	2.41	Hookeroside D-isomer (**84**)
1305.6489	41.03	2.33	Sapindoside G (**77**)
447.0931	7.67	1.52	Isoorlentin (**22**)
461.1087	8.29	1.40	Isoscoparin (**23**)
445.1134	9.68	1.35	Swertisin (**30**)
487.3423	36.78	1.13	Euscaphic acid (**72**)
747.2717	12.84	1.06	Sylvestroside I (**35**)
515.1190	11.04	1.05	Isochlorogenic acid A (**33**)

## Data Availability

The original contributions presented in this study are included in this article. Further inquiries can be directed to the corresponding authors.

## Supplementary Materials

The following supporting information can be downloaded at: https://www.mdpi.com/article/10.3390/molecules31111868/s1, Figure S1: The base peak ion chromatogram of *P. hookeri* in the positive ion mode; Figure S2: Chemical structures of iridoids and phenylpropanoids identified; Figure S3: Chemical structures of triterpenes identified; Figure S4: Chemical structures of other types of identified; Figure S5: The FBMN diagrams of the aboveground and underground parts of *P. hookeri* in the automatic extraction mode; Figure S6: The FBMN diagrams of the aboveground and underground parts of *P. hookeri* with response values greater than 1000; Figure S7: The FBMN diagrams of the aboveground and underground parts of *P. hookeri* with response values greater than 5000; Figure S8: The FBMN diagrams of the aboveground and underground parts of *P. hookeri* with response values greater than 10,000; Figure S9: The FBMN diagrams of the aboveground and underground parts of *P. hookeri* with response values greater than 50,000; Figure S10: Multivariate statistical results of the aboveground and underground parts of *P. hookeri* in the automatic extraction mode. (A) PCA score plot, (B) OPLS-DA score plot, (C) OPLS-DA loadings plot, (D) Replacement test plot; Figure S11: Multivariate statistical results of the aboveground and underground parts of *P. hookeri* with response values greater than 1000. (A) PCA score plot, (B) OPLS-DA score plot, (C) OPLS-DA loadings plot, (D) Replacement test plot; Figure S12: Multivariate statistical results of the aboveground and underground parts of *P. hookeri* with response values greater than 5000. (A) PCA score plot, (B) OPLS-DA score plot, (C) OPLS-DA loadings plot, (D) Replacement test plot; Figure S13: Multivariate statistical results of the aboveground and underground parts of *P. hookeri* with response values greater than 10,000. (A) PCA score plot, (B) OPLS-DA score plot, (C) OPLS-DA loadings plot, (D) Replacement test plot; Figure S14: Multivariate statistical results of the aboveground and underground parts of *P. hookeri* with response values greater than 50,000. (A) PCA score plot, (B) OPLS-DA score plot, (C) OPLS-DA loadings plot, (D) Replacement test plot; Table S1: MS data for compounds identified from *P. hookeri* in positive ion mode.

## Abbreviations

The following abbreviations are used in this manuscript:

BBMN	Block-based molecular networking
CLMN	Classical molecular networking
DDA	Data-dependent acquisition
DIA	Data-independent acquisition
FBMN	Feature-based molecular networking
GNPS	Global Natural Product Social
IIMN	Ion identity molecular networking
OPLS-DA	Orthogonal partial least square-discriminant analysis
PAD	Paper-based analytical device
PCA	Principal component analysis
RA	Rheumatoid arthritis
