# Supplementary material for: Chemical Profiling of Aboveground and Underground Parts of Pterocephalus hookeri by Integrated FBMN, Untargeted LC-MS Metabolomics, and PAD-DESI-MSI
Source: Molecules. 2026 May 29;31(11):1868. doi: 10.3390/molecules31111868 (PMC13258631; doi:10.3390/molecules31111868)
Supplement: Supplementary file 1 [file molecules-31-01868-s001.zip › molecules-4321124-supplementary.pdf]

Supporting Information

Chemical Profiling of Aboveground and Underground Parts of *Pterocephalus hookeri* by Integrated FBMN, Untargeted LC-MS Metabolomics, and PAD-DESI-MSI

Jiaxing Luo [1], Lanlan Fang [1], Muze Yu [1], Di Yang [1], Jing Zhang [1], Jia Yu [1], Ce Tang [1], Tingting Kuang [1,*]

[a] *Lab for Innovation & Effective Uses of Chinese Drug Germplasm Resources, School of Ethnic Medicine, Chengdu University of Traditional Chinese Medicine, Chengdu, 611137, China*

[*]Corresponding authors:

Tingting Kuang

Lab for Innovation & Effective Uses of Chinese Drug Germplasm Resources, Chengdu University of Traditional Chinese Medicine, Wenjiang campus: 1166 Liutai Avenue, Wenjiang District, Chengdu, Sichuan, China 611137

E-mail addresses: kuangtingting@cdutcm.edu.cn

Legends

Figure. S1. The base peak ion chromatogram of *P. hookeri* in the positive ion mode.

Figure. S2. Chemical structures of iridoids and phenylpropanoids identified.

Figure. S3. Chemical structures of triterpenes identified.

Figure. S4. Chemical structures of other types of identified.

Figure. S5. The FBMN diagrams of the aboveground and underground parts of *P. hookeri* in the automatic extraction mode.

Figure. S6. The FBMN diagrams of the aboveground and underground parts of *P. hookeri* with response values greater than 1000.

Figure. S7. The FBMN diagrams of the aboveground and underground parts of *P. hookeri* with response values greater than 5000.

Figure. S8. The FBMN diagrams of the aboveground and underground parts of *P. hookeri* with response values greater than 10,000.

Figure. S9. The FBMN diagrams of the aboveground and underground parts of *P. hookeri* with response values greater than 50,000.

Figure. S10. Multivariate statistical results of the aboveground and underground parts of *P. hookeri* in the automatic extraction mode. (A) PCA score plot, (B) OPLS-DA score plot, (C) OPLS-DA loadings plot, (D) Replacement test plot.

Figure. S11. Multivariate statistical results of the aboveground and underground parts of P. hookeri with response values greater than 1000. (A) PCA score plot, (B) OPLS-DA score plot, (C) OPLS-DA loadings plot, (D) Replacement test plot.

Figure. S12. Multivariate statistical results of the aboveground and underground parts of P. hookeri with response values greater than 5000. (A) PCA score plot, (B) OPLS-DA score plot, (C) OPLS-DA loadings plot, (D) Replacement test plot.

Figure. S13. Multivariate statistical results of the aboveground and underground parts of P. hookeri with response values greater than 10,000. (A) PCA score plot, (B) OPLS-DA score plot, (C) OPLS-DA loadings plot, (D) Replacement test plot.

Figure. S14. Multivariate statistical results of the aboveground and underground parts of P. hookeri with response values greater than 50,000. (A) PCA score plot, (B) OPLS-DA score plot, (C) OPLS-DA loadings plot, (D) Replacement test plot.

Table S1. MS data for compounds identified from *P. hookeri* in positive ion mode.

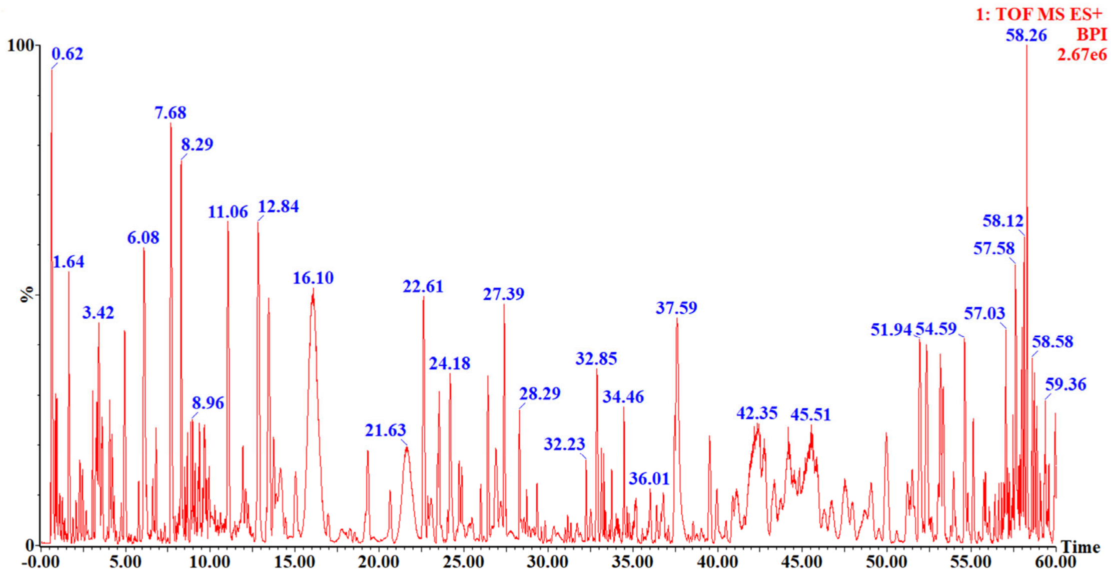

Figure. S1. The base peak ion chromatogram of *P. hookeri* in positive ion mode.

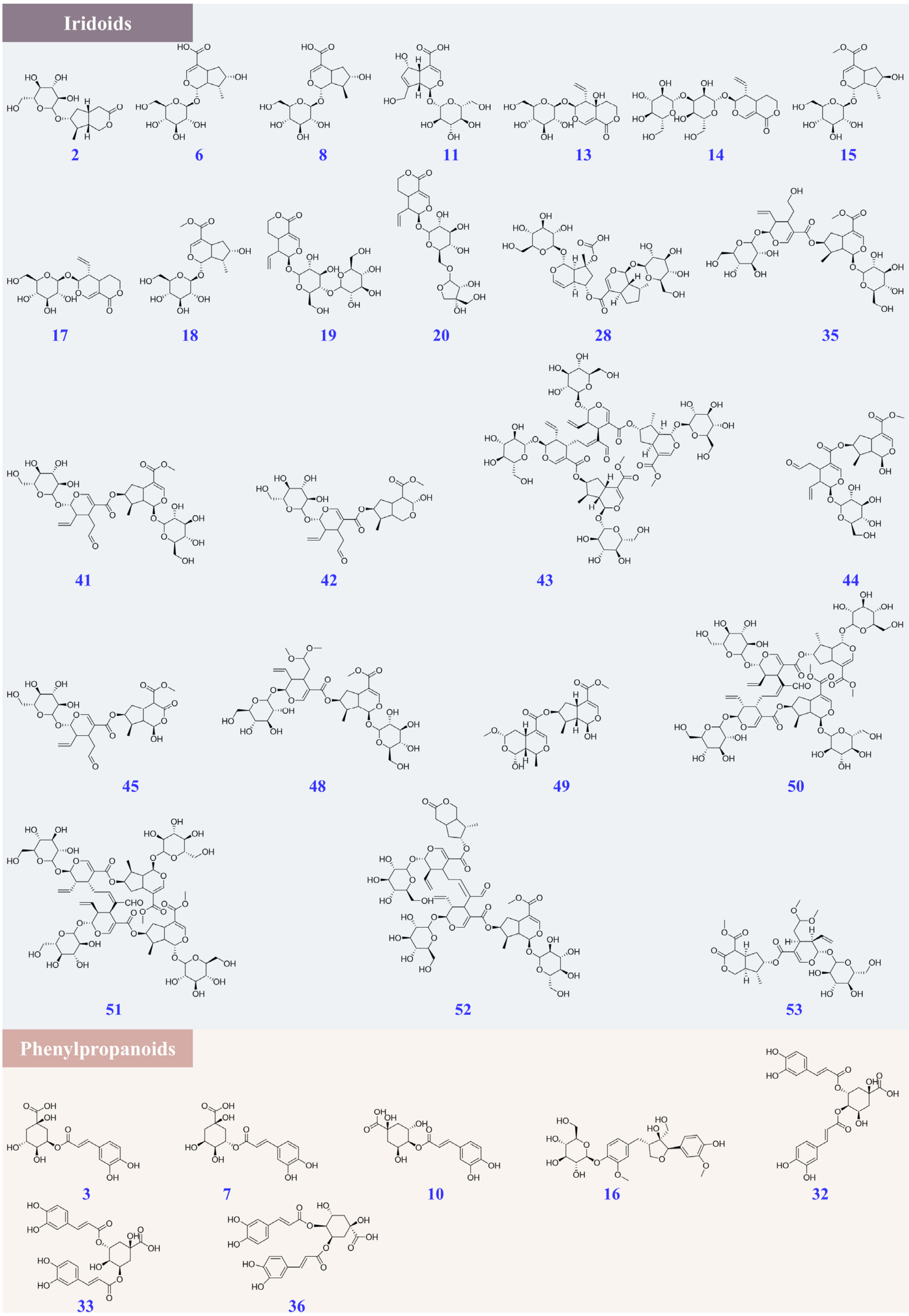

Figure. S2. Chemical structures of iridoids and phenylpropanoids identified.

Triterpenes

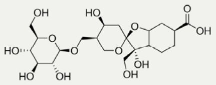

4

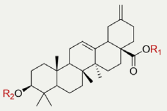

66 R_1=Ara[(3,1)Ara](2,1)Rha, R_2=Glc(6,1)Glc(4,1)Rha

70 R_1=Xyl(3,1)Glc[(2,1)Rha](4,1)Rha(4,1)Xyl, R_2=Glc

71 R_1=Xyl(3,1)Glc[(2,1)Rha](4,1)Rha, R_2=Glc

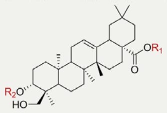

67 R_1=Ara(2,1)Rha(3,1)Xyl, R_2=Glc(6,1)Glc

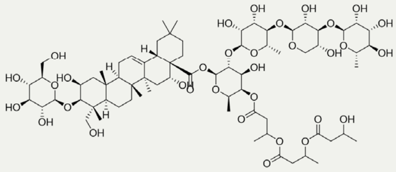

69

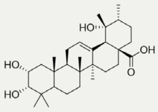

72

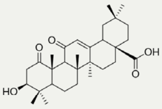

75

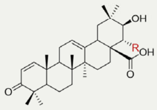

76 R=OH

91 R=H

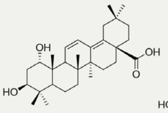

87

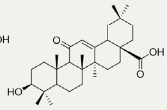

89

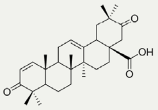

92

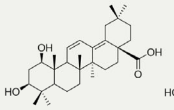

94

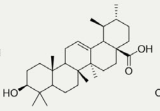

98

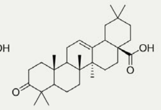

99

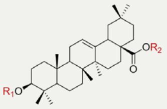

57 R_1=Xyl(2,1)Rha(3,1)Xyl(4,1)Glc(4,1)Xyl(3,1)Rha, R_2=Glc(6,1)Glc

58 R_1=Xyl(2,1)Rha(3,1)Xyl(4,1)Glc(4,1)Xyl(2,1)Rha, R_2=Glc(6,1)Glc

59 R_1=Xyl(2,1)Rha(3,1)Xyl(4,1)Glc(4,1)Xyl, R_2=Glc(6,1)Glc

60 R_1=Xyl(2,1)Rha(3,1)Xyl(4,1)Glc, R_2=Glc(6,1)Glc

62 R_1=Ara(2,1)Rha(3,1)Xyl, R_2=Glc(6,1)Glc

63 R_1=Rha(2,1)Ara, R_2=Glc(6,1)Glc

65 R_1=Glc(2,1)Xyl, R_2=Glc(2,1)Xyl[(2,1)Glc](3,1)Xyl

68 R_1= Xyl(3,1)Rha(3,1)Xyl, R_2=H

73 R_1= Xyl(4,1)Glc, R_2=Xyl(4,1)Rha(3,1)Xyl

74 R_1=Xyl(3,1)Rha(3,1)Xyl(3,1)Xyl, R_2=Glc(6,1)Glc

77 R_1=Xyl(2,1)Rha(3,1)Xyl(3,1)Glc(6,1)Xyl(2,1)Rha, R_2=H

78 R_1=Xyl(2,1)Rha(3,1)Xyl(3,1)Glc(4,1)Xyl, R_2=H

79 R_1=Xyl(2,1)Rha(3,1)Xyl(4,1)Glc(4,1)Xyl, R_2=H

80 R_1=Xyl(2,1)Rha(3,1)Xyl(3,1)Glc, R_2=H

81 R_1=Xyl(2,1)Glc(3,1)Xyl(3,1)Rha, R_2=Glc(6,1)Glc

82 R_1=Xyl(2,1)Rha(3,1)Xyl(4,1)Glc(4,1)Xyl(2,1)Rha, R_2=H

83 R_1=Xyl(2,1)Rha(3,1)Xyl(3,1)Rha(3,1)Xyl(3,1)Xyl, R_2=H

84 R_1=Xyl[(2,1)Xyl](3,1)Rha(3,1)Xyl(3,1)Rha(3,1)Xyl, R_2=H

85 R_1=Xyl(2,1)Rha(3,1)Xyl(3,1)Rha, R_2=H

86 R_1=Xyl(2,1)Rha(3,1)Xyl, R_2=H

88 R_1=Xyl(2,1)Rha, R_2=H

93 R_1=Xyl, R_2=H

97 R_1=H, R_2=H

Figure. S3. Chemical structures of triterpenes identified.

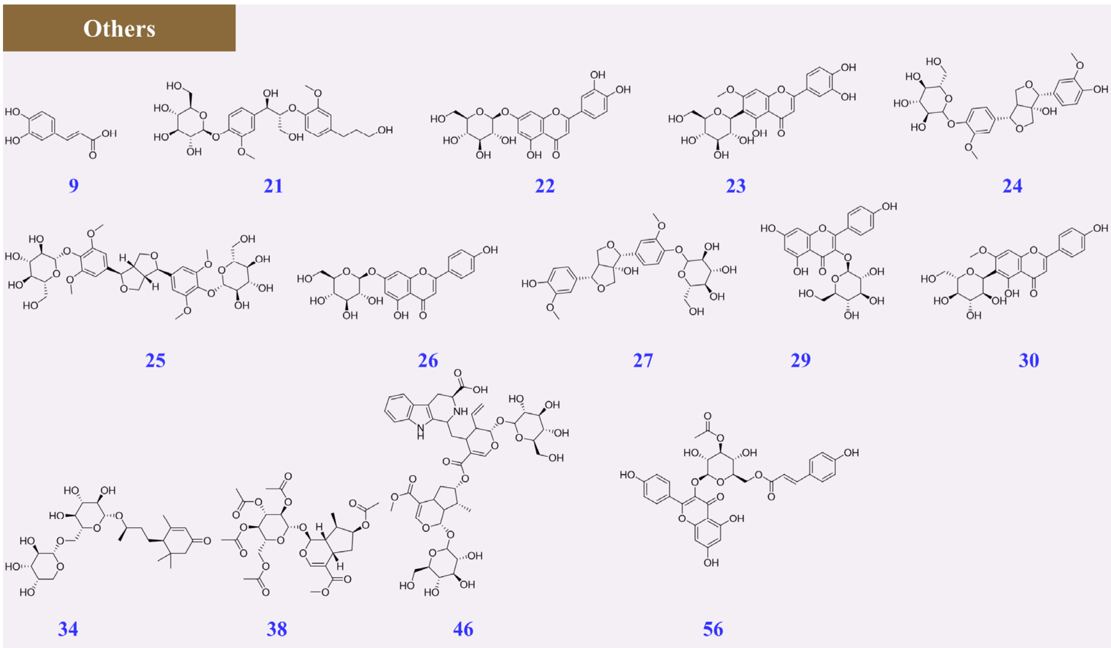

Figure. S4. Chemical structures of other types of identified.

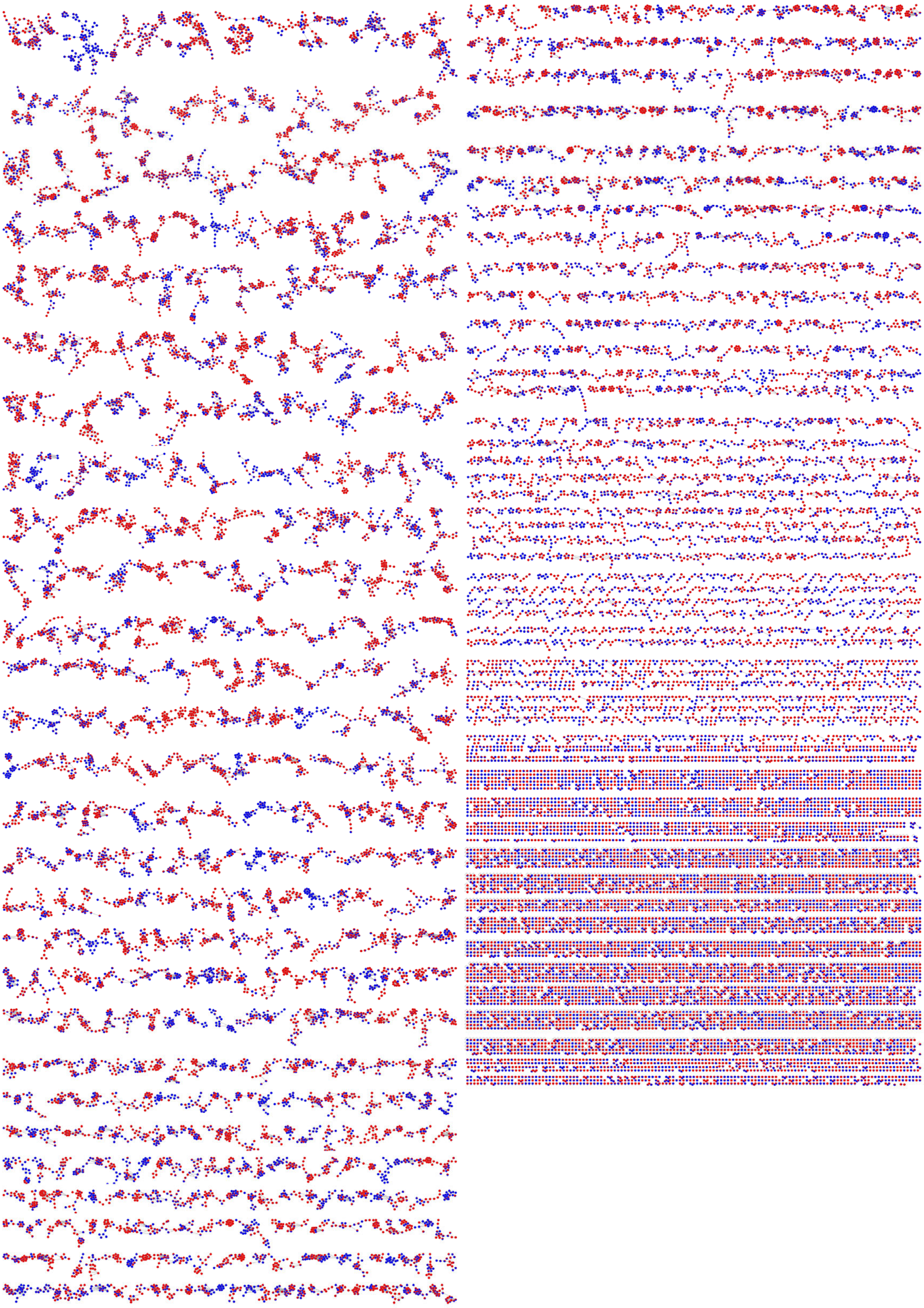

Figure. S5. The FBMN diagrams of the aboveground and underground parts of *P. hookeri* in the automatic extraction mode.

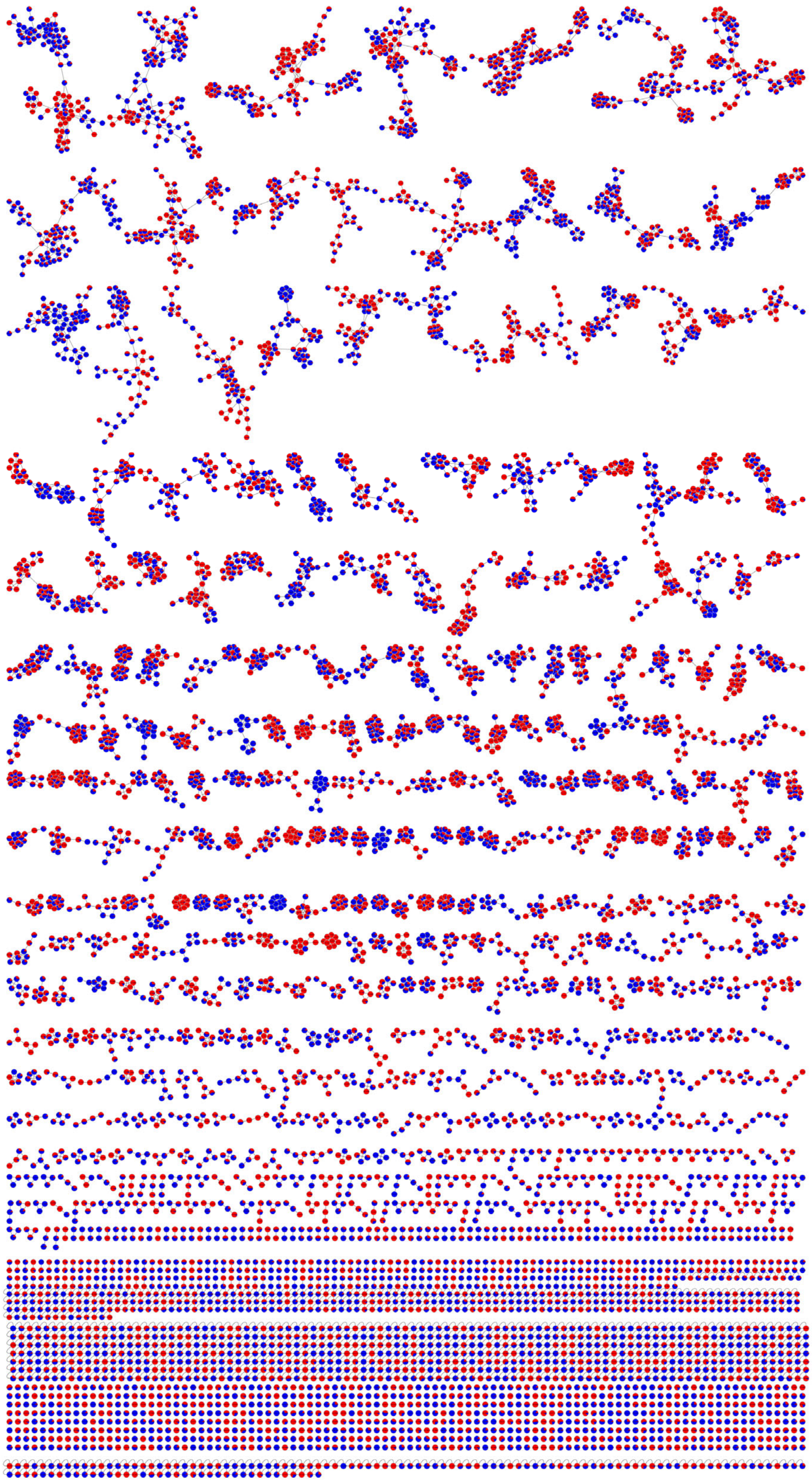

Figure. S6. The FBMN diagrams of the aboveground and underground parts of *P. hookeri* with response values greater than 1000.

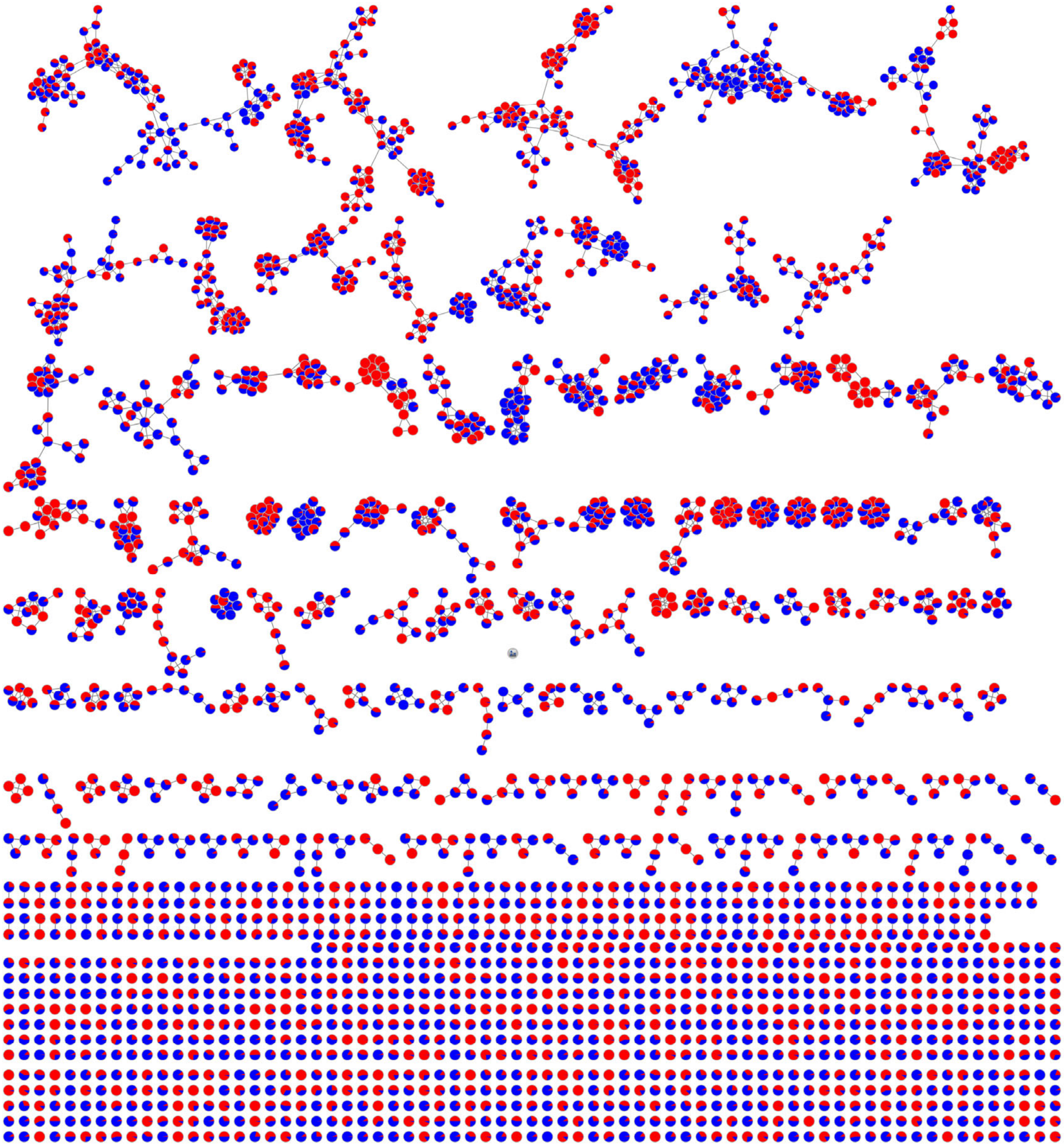

Figure. S7. The FBMN diagrams of the aboveground and underground parts of *P. hookeri* with response values greater than 5000.

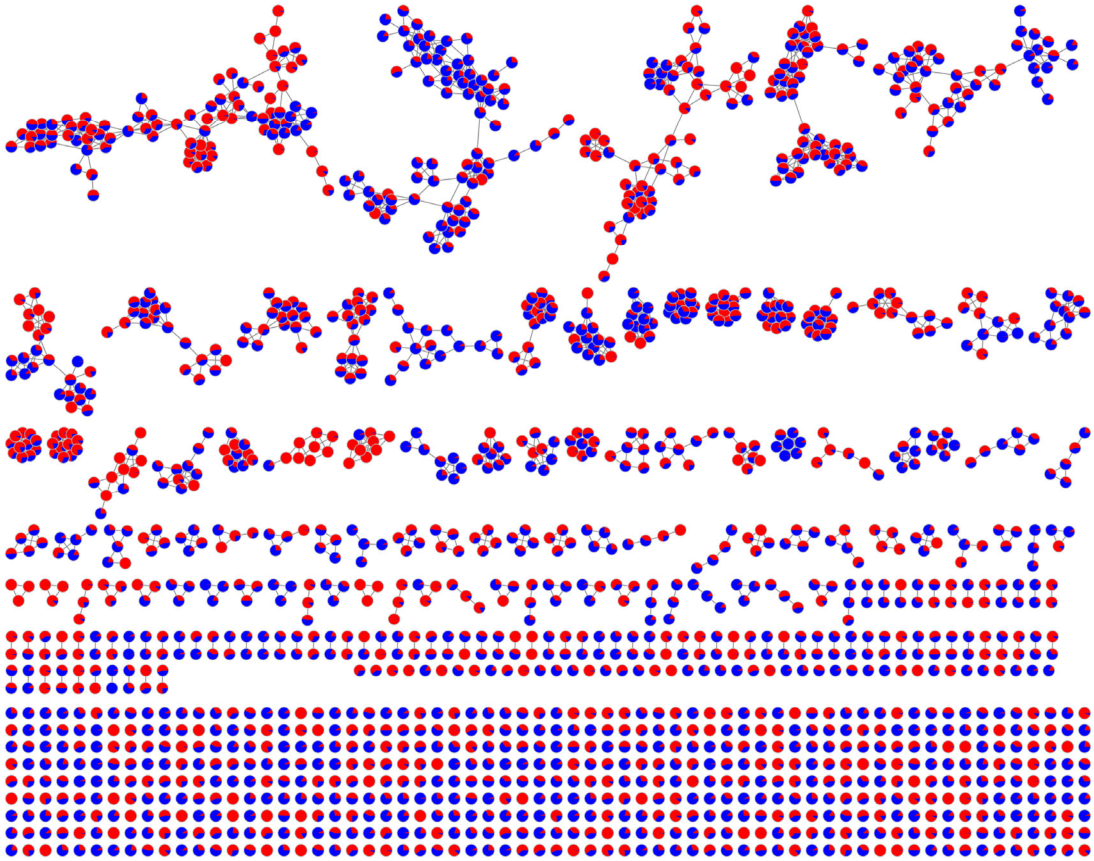

Figure. S8. The FBMN diagrams of the aboveground and underground parts of *P. hookeri* with response values greater than 10,000.

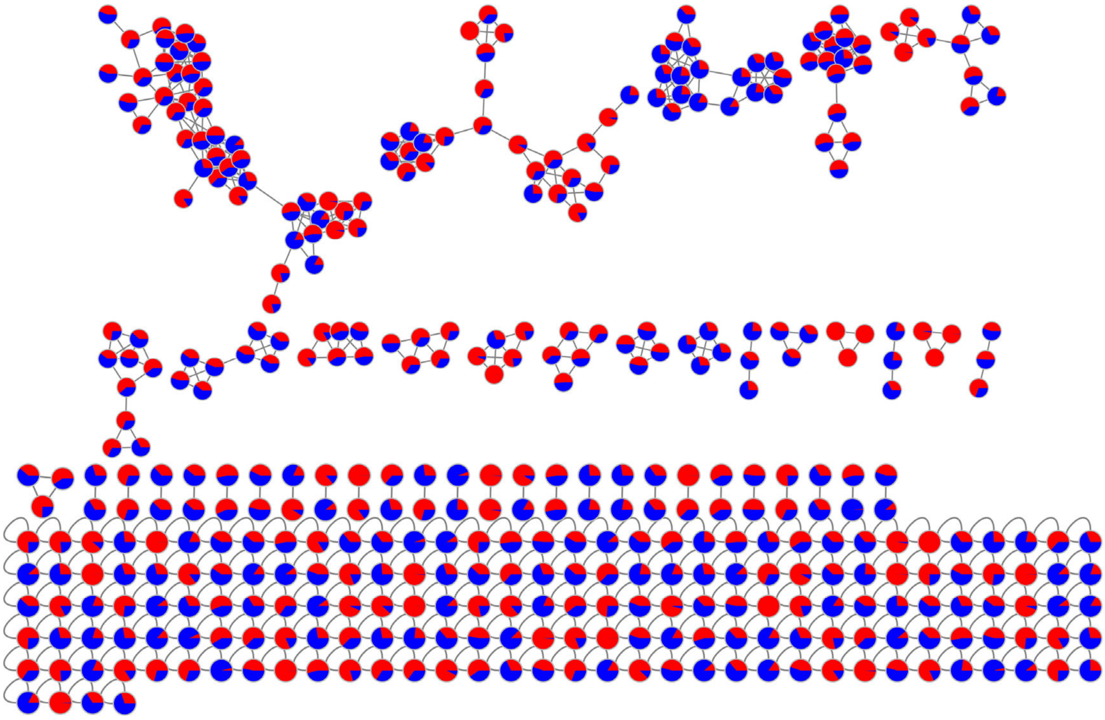

Figure. S9. The FBMN diagrams of the aboveground and underground parts of *P. hookeri* with response values greater than 50,000.

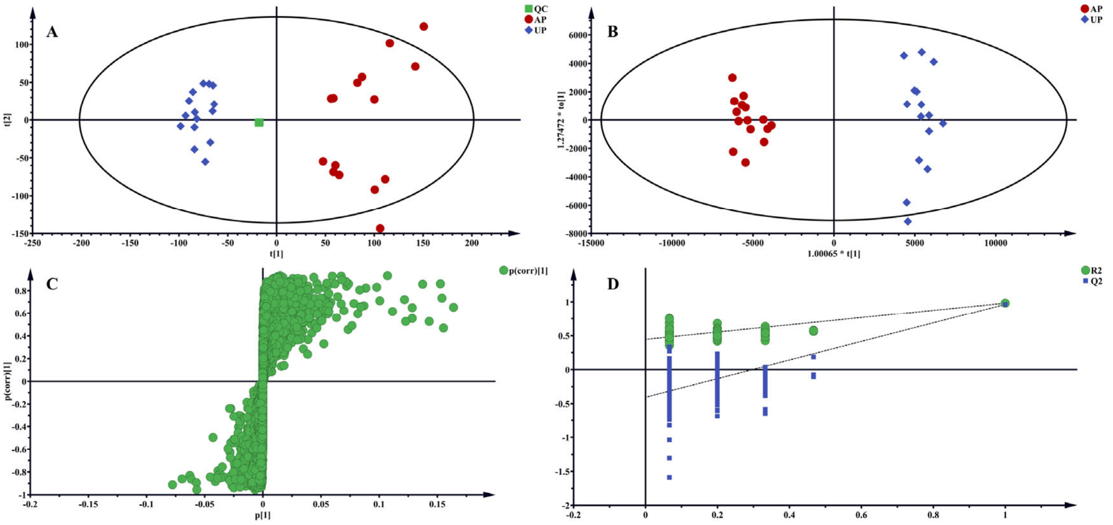

Figure. S10. Multivariate statistical results of the aboveground and underground parts of *P. hookeri* in the automatic extraction mode. (A) PCA score plot, (B) OPLS-DA score plot, (C) OPLS-DA loadings plot, (D) Replacement test plot.

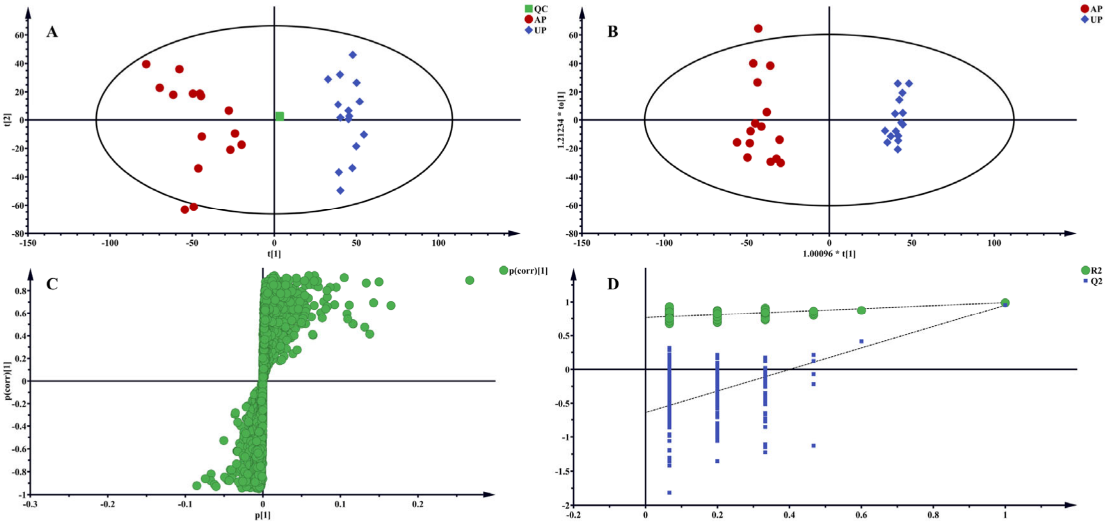

Figure. S11. Multivariate statistical results of the aboveground and underground parts of P. hookeri with response values greater than 1000. (A) PCA score plot, (B) OPLS-DA score plot, (C) OPLS-DA loadings plot, (D) Replacement test plot.

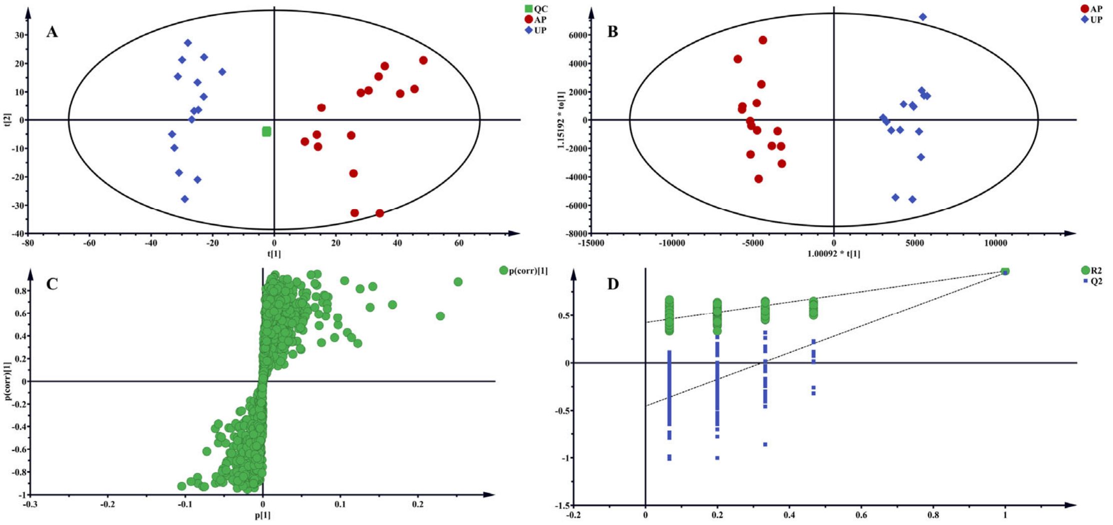

Figure. S12. Multivariate statistical results of the aboveground and underground parts of P. hookeri with response values greater than 5000. (A) PCA score plot, (B) OPLS-DA score plot, (C) OPLS-DA loadings plot, (D) Replacement test plot.

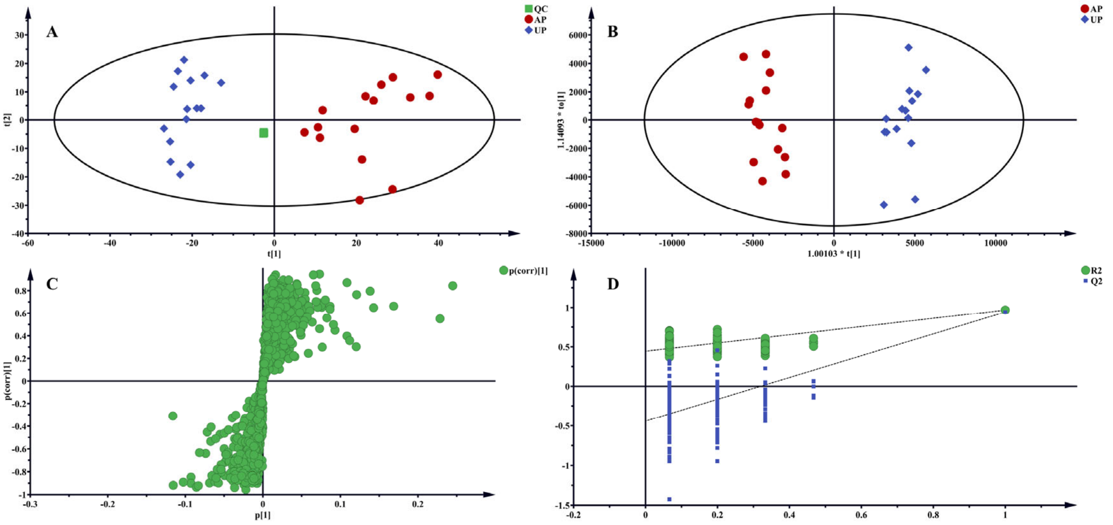

Figure. S13. Multivariate statistical results of the aboveground and underground parts of P. hookeri with response values greater than 10,000. (A) PCA score plot, (B) OPLS-DA score plot, (C) OPLS-DA loadings plot, (D) Replacement test plot.

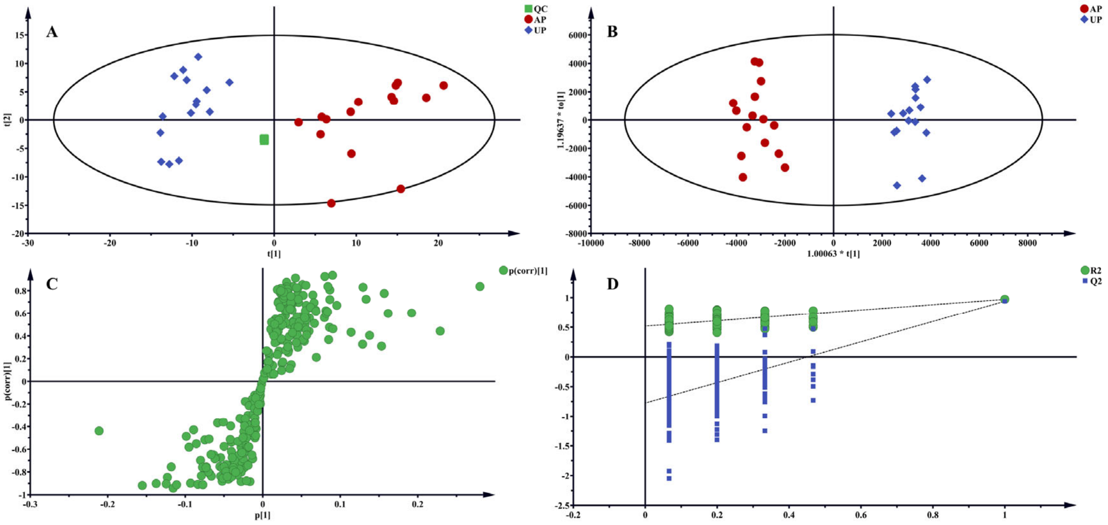

Figure. S14. Multivariate statistical results of the aboveground and underground parts of P. hookeri with response values greater than 50,000. (A) PCA score plot, (B) OPLS-DA score plot, (C) OPLS-DA loadings plot, (D) Replacement test plot.

Table S1. MS data for compounds identified from P. hookeri in positive ion mode.

No.	T_R （min）	Identification	Formula	MS/[M-H]$^-$	δ （ppm）	Fragment ions
1	1.58	Unknown 1	$C_{19}H_{20}O_{10}$	407.1553	1.5	
2	2.11	Ptehoside B	$C_{15}H_{24}O_8$	331.1388	−1.5	687.2842 [2M+Na]$^+$, 665.3022 [2M+H]$^+$, 355.1370 [M+Na]$^+$, 333.1551 [M+H]$^+$
3	2.24	Neochlorogenic acid	$C_{16}H_{18}O_9$	353.0872	−0.3	163.0399 [M+H−caffeoyl]$^+$, 145.0292 [M+H−caffeoyl−H_2O]$^+$
4	2.47	Phyllaemblicin D	$C_{21}H_{34}O_{13}$	493.1920	−0.2	517.1907 [M+Na]$^+$
5	2.60	Unknown 2	$C_{20}H_{32}O_{12}$	463.1813	−0.6	929.2616 [2M+H]$^+$, 487.1794 [M+Na]$^+$
6	3.24	Loganic acid	$C_{16}H_{24}O_{10}$	375.13079	4.8	755.2890 [2M+Na]$^+$, 753.2825 [2M+H]$^+$, 399.1268 [M+Na]$^+$, 359.1347 [M+H−H_2O]$^+$, 215.0921 [M+H−Glc]$^+$, 197.0817 [M+H−Glc−H_2O]$^+$, 179.0714 [M+H−Glc−2H_2O]$^+$, 151.0762 [M+H−Glc−2H_2O−CO]$^+$, 161.0606 [M+H−Glc−3H_2O]$^+$, 133.0653 [M+H−Glc−3H_2O−CO]$^+$
7	3.60	Chlorogenic acid	$C_{16}H_{18}O_9$	353.0877	1.1	731.2487 [2M+Na+H]$^+$, 709.1887 [2M+H]$^+$, 377.0846 [M+Na]$^+$, 163.0402 [M+H−caffeoyl]$^+$, 145.9304 [M+H−caffeoyl−H_2O]$^+$
8	3.95	8-Epiloganic acid	$C_{16}H_{24}O_{10}$	375.1292	0.3	215.0923 [M+H−Glc]$^+$,197.0814 [M+H−Glc−H_2O]$^+$, 179.0712 [M+H−Glc−H_2O−H_2O]$^+$, 161.0603 [M+H−Glc−H_2O−H_2O−H_2O]$^+$, 151.0759 [M+H−Glc−H_2O−H_2O−CO]$^+$, 133.0653 [M+H−Glc−H_2O−H_2O−CO−H_2O]$^+$
9	4.06	Caffeic acid	$C_9H_8O_4$	179.0347	1.7	181.0503 [M+H]$^+$, 163.0400 [M+H−H_2O]$^+$, 145.0291 [M+H−2H_2O]$^+$
10	4.11	Cryptochlorogenic acid	$C_{16}H_{18}O_9$	353.0878	1.4	
11	4.31	Deacetylasperulosidic acid	$C_{16}H_{22}O_{11}$	389.1083	−0.3	413.1060 [M+Na]$^+$
12	4.41	Unkonwn 3	$C_{32}H_{22}O_4$	469.1452	2.6	941.3166 [2M+H]$^+$, 471.1615 [M+H]$^+$
13	4.69	Swertimarin	$C_{16}H_{22}O_{10}$	373.1133	−0.5	213.0765 [M+H−Glc]$^+$, 151.0396 [M+H−Glc−H_2O−H_2O−C_2H_2]$^+$, 125.0241 [M+H−Glc−H_2O−C_4H_6O]$^+$

14	4.89	3′-*O*-β-D-glucopyranosyl sweroside	$C_{22}H_{32}O_{14}$	519.1717	0.6	1063.3470 $[2M+Na]^+$, 1041.33566 $[2M+H]^+$, 543.1692 $[M+Na]^+$, 359.1345 $[M+H-Glc]^+$, 197.0815 $[M+H-2Glc]^+$, 179.0710 $[M+H-2Glc-H_2O]^+$, 127.0396 $[M+H-2Glc-C_4H_6O]^+$
15	5.57	7-Epiloganin	$C_{17}H_{26}O_{10}$	389.1448	0.0	229.1077 $[M+H-Glc]^+$, 211.0972 $[M+H-Glc-H_2O]^+$, 197.0825 $[M+H-Glc-CH_3OH]^+$, 179.0704 $[M+H-Glc-H_2O-CH_3OH]^+$, 151.0756 $[M+H-Glc-H_2O-CH_3OH-CO]^+$, 161.0606 $[M+H-Glc-H_2O-CH_3OH-H_2O]^+$, 133.0651 $[M+H-Glc-H_2O-CH_3OH-CO-H_2O]^+$
16	5.76	Berchemol 4′-*O*-β-D-glucoside	$C_{26}H_{34}O_{12}$	537.1971	−0.2	561.1949 $[M+Na]^+$
17	6.06	Sweroside	$C_{16}H_{22}O_9$	357.1181	−1.4	739.2435 $[2M+Na+H]^+$, 717.2612 $[2M+H]^+$, 381.1165 $[M+Na]^+$, 197.0827 $[M+H-Glc]^+$, 179.0714 $[M+H-Glc-H_2O]^+$, 151.0762 $[M+H-Glc-H_2O-CO]^+$, 127.0399 $[M+H-Glc-C_4H_6O]^+$
18	6.20	Loganin	$C_{17}H_{26}O_{10}$	413.1422/$[M+Na]^+$	−0.5	803.2963 $[2M+Na]^+$, 229.1080 $[M+H-Glc]^+$, 211.0972 $[M+H-Glc-H_2O]^+$, 197.0818 $[M+H-Glc-CH_3OH]^+$, 179.0713 $[M+H-Glc-H_2O-CH_3OH]^+$, 151.0761 $[M+H-Glc-H_2O-CH_3OH-CO]^+$
19	6.59	Dipsanoside H	$C_{22}H_{32}O_{14}$	519.1720	1.2	1063.3507 $[2M+Na]^+$, 1041.3677 $[2M+H]^+$, 359.1350 $[M+H-Glc]^+$, 197.0817 $[M+H-Glc-Glc]^+$, 179.0711 $[M+H-Glc-Glc-H_2O]^+$, 127.0397 $[M+H-Glc-Glc-C_4H_6O]^+$
20	6.78	6'-*O*-beta-Apiofuranosylsweroside	$C_{21}H_{30}O_{13}$	489.1613	1.0	1003.3285 $[2M+Na]^+$, 981.3466 $[2M+H]^+$, 513.1582 $[M+Na]^+$, 359.1346 $[M+H-Api]^+$, 197.0817 $[M+H-Api-Glc]^+$, 179.0712 $[M+H-Api-Glc-H2O]^+$, 151.0760 $[M+H-Api-Glc-CO]^+$, 127.0397 $[M+H-Api-Glc-C_4H_6O]^+$

21	7.09	(7R,8S)-erythro-7,9,9'-trihydroxy-3,3'-dimethoxy-8-*O*-4'-neolignan-4-*O*-β-D-glucoside	$C_{26}H_{36}O_{12}$	539.2133	0.7	563.2101 $[M+Na]^+$
22	7.69	Isoorlentin	$C_{21}H_{20}O_{11}$	447.0926	−0.2	449.1091 $[M+H]^+$
23	8.29	Isoscoparin	$C_{22}H_{22}O_{11}$	461.1094	2.2	463.1255 $[M+H]^+$
24	8.92	8-Hydroxypinoresinol-4′-*O*-β-D-glucopyranoside	$C_{26}H_{32}O_{12}$	535.1816	0.0	1095.3713 $[2M+Na]^+$, 559.1791 $[M+Na]^+$
25	9.04	(-)-Syringaresinol diglucoside	$C_{34}H_{46}O_{18}$	741.2637	0.1	765.2592 $[M+Na]^+$, 743.2674 $[M+H]^+$
26	9.10	Apigenin 7-*O*-glucoside	$C_{21}H_{20}O_{10}$	431.0973	−1.2	455.0949 $[M+Na]^+$, 433.1138 $[M+H]^+$
27	9.16	8'-Hydroxylpinoresionl-4-*O*-β-D-glucoside	$C_{26}H_{32}O_{12}$	535.1818	0.4	559.1796 $[M+Na]^+$
28	9.54	Caryocanoside B	$C_{32}H_{46}O_{19}$	733.2548	−1.0	1469.5359 $[2M+H]^+$, 757.2537 $[M+Na]^+$, 735.2712 $[M+H]^+$
29	9.61	Astragalin	$C_{21}H_{20}O_{11}$	447.0928	0.2	449.1084 $[M+H]^+$, 287.0555 $[M+H-Glc]^+$
30	9.68	Swertisin	$C_{22}H_{22}O_{10}$	445.1134	−0.2	447.1296 $[M+H]^+$
31	10.63	Unknown 4	$C_{26}H_{28}O_{14}$	563.1398	−0.5	587.1366 $[M+Na]^+$, 565.1570 $[M+H]^+$
32	10.84	Isochlorogenic acid B	$C_{25}H_{24}O_{12}$	515.1190	0.0	1055.4712 $[2M+Na]^+$, 1033.2632 $[2M+H]^+$, 499.1244 $[M+H-H_2O]^+$, 337.2446 $[M+H-\text{caffeic acid}]^+$, 163.0401 $[\text{caffeic acid}-H_2O+H]^+$, 145.0296 $[\text{caffeic acid}-H_2O-H_2O+H]^+$
33	11.04	Isochlorogenic acid A	$C_{25}H_{24}O_{12}$	515.1209	3.7	1055.2448 $[2M+Na]^+$, 539.1166 $[M+Na]^+$, 499.1260 $[M+H-H_2O]^+$, 337.0930 $[M+H-\text{caffeic acid}]^+$, 163.0401 $[\text{caffeic acid}-H_2O+H]^+$, 145.0294 $[\text{caffeic acid}-H_2O-H_2O+H]^+$
34	12.25	Foliasalacioside B_1	$C_{24}H_{40}O_{11}$	503.2509	3.4	1009.3946 $[2M+H]^+$, 527.2467 $[M+Na]^+$, 505.2652 $[M+H]^+$

35	12.84	Sylvestroside I	$C_{33}H_{48}O_{19}$	747.2712	0.0	1497.5684 [2M+H]$^+$, 587.2351 [M+H−Glc]$^+$, 569.2244 [M+H−Glc−H_2O]$^+$, 551.2133 [M+H−Glc−2H_2O]$^+$, 425.1820 [M+H−2Glc]$^+$, 407.1708 [M+H−2Glc−H_2O]$^+$, 389.1602 [M+H−2Glc−2H_2O]$^+$, 197.0828 [M+H−2Glc−$C_{11}H_{15}O_5$]$^+$, 179.0713 [M+H−2Glc−$C_{11}H_{15}O_5$−H_2O]$^+$, 151.0763 [M+H−2Glc−$C_{11}H_{15}O_5$−H_2O−CO]$^+$
36	13.41	Isochlorogenic acid C	$C_{25}H_{24}O_{12}$	515.1215	4.9	1055.2892 [2M+Na]$^+$, 539.1164 [M+Na]$^+$, 499.1246 [M+H−H_2O]$^+$, 337.0925 [M+H−caffeic acid]$^+$, 163.0402 [caffeic acid−H_2O+H]$^+$, 145.0292 [caffeic acid−2H_2O+H]$^+$
37	13.71	Unknown 5	$C_{25}H_{36}O_{12}$	527.2136	1.3	1057.4503 [2M+H]$^+$, 551.2106 [M+Na]$^+$, 529.2291 [M+H]$^+$
38	14.16	Loganin pentaacetate	$C_{27}H_{36}O_{15}$	599.1979	0.5	1201.4073 [2M+H]$^+$, 623.1955 [M+Na]$^+$, 601.2120 [M+H]$^+$
39	14.76	Laciniatoside I-isomer	$C_{27}H_{38}O_{14}$	585.2181	−0.3	609.2161 [M+Na]$^+$
40	15.02	Laciniatoside I-isomer	$C_{27}H_{38}O_{14}$	585.2182	−0.2	609.2165 [M+Na]$^+$
41	16.11	Cantleyoside	$C_{33}H_{46}O_{19}$	745.2563	1.1	1515.5183 [2M+Na]$^+$, 769.2534 [M+Na]$^+$, 585.2196 [M+H−Glc]$^+$, 423.1655 [M+H−Glc−Glc]$^+$, 213.0768 [$C_{10}H_{12}O_5$+H]$^+$, 211.0972 [$C_{11}H_{14}O_4$+H]$^+$, 195.0659 [$C_{10}H_{12}O_5$+H−H_2O]$^+$, 193.0865 [$C_{11}H_{14}O_4$+H−H_2O]$^+$
42	17.75	Laciniatoside I	$C_{27}H_{38}O_{14}$	585.2175	1.4	1195.4436 [2M+Na]$^+$, 609.2162 [M+Na]$^+$, 587.2341 [M+H]$^+$, 425.1808 [M+H−Glc]$^+$, 389.1594 [M+H−Glc−2H_2O]$^+$, 197.0815 [M+H−Glc−2H_2O−$C_{10}H_8O_4$]$^+$
43	18.24	Strychoside B	$C_{66}H_{90}O_{37}$	1473.5085	0.1	1497.5070 [M+Na]$^+$
44	21.41	Sylvestroside III	$C_{27}H_{36}O_{14}$	583.2031	0.7	1191.4110 [2M+Na]$^+$, 607.2003 [M+Na]$^+$, 423.1651 [M+H−Glc]$^+$, 213.0766 [M+H−Glc−$C_{11}H_{14}O_4$]$^+$, 195.0659 [M+H−Glc−$C_{11}H_{14}O_4$−H_2O]$^+$, 179.0710 [M+H−Glc−$C_{11}H_{14}O_4$−2H_2O]$^+$, 151.0395 [M+H−Glc−$C_{11}H_{14}O_4$−2H_2O−CO]$^+$

45	21.54	Sylvestroside IV	$C_{27}H_{36}O_{14}$	583.2037	1.7	1191.4117 [2M+Na]$^+$, 607.2004 [M+Na]$^+$, 423.1652 [M+H−Glc]$^+$, 213.0766 [M+H−Glc−$C_{11}H_{14}O_4$]$^+$, 195.0659 [M+H−Glc−$C_{11}H_{14}O_4$−H_2O]$^+$, 179.0710 [M+H−Glc−$C_{11}H_{14}O_4$−2H_2O]$^+$, 151.0395 [M+H−Glc−$C_{11}H_{14}O_4$−2H_2O−CO]$^+$
46	22.27	Pterocephaline	$C_{44}H_{56}N_2O_{20}$	931.3361	−1.8	933.3694 [M+H]$^+$, 771.2975[M+H−Glc]$^+$, 609.2109 [M+H−2Glc]$^+$
47	22.84	Unknown 6	$C_{53}H_{72}O_{30}$	1187.4044	1.2	1211.4016 [M+Na]$^+$, 1189.4197 [M+H]$^+$, 1027.3673 [M+H−Glc]$^+$, 865.3144 [M+H−2Glc]$^+$, 703.2607 [M+H−3Glc]$^+$
48	24.15	Triplostoside A	$C_{35}H_{52}O_{20}$	791.2966	−1.0	
49	25.35	Pterocenoids C	$C_{22}H_{30}O_{10}$	453.1759	−0.4	477.1737 [M+Na]$^+$
50	25.95	Dipsanoside B	$C_{66}H_{90}O_{37}$	1473.5093	0.7	1313.4736 [M+H−Glc]$^+$, 1295.4628 [M+H−Glc−H_2O]$^+$, 1133.4095 [M+H−2Glc−H_2O]$^+$, 971.3563 [M+H−3Glc−H_2O]$^+$, 809.3029 [M+H−4Glc−H_2O]$^+$, 791.2920 [M+H−4Glc−2H_2O]$^+$
51	26.39	Dipsanoside A	$C_{66}H_{90}O_{37}$	1473.5087	0.3	1313.4473 [M+H−Glc]$^+$, 1295.4628 [M+H−Glc−H_2O]$^+$, 1151.4205 [M+H−2Glc]$^+$, 1133.4098 [M+H−2Glc−H_2O]$^+$, 971.3578 [M+H−3Glc−H_2O]$^+$, 809.3026 [M+H−4Glc−H_2O]$^+$, 791.2925 [M+H−4Glc−2H_2O]$^+$
52	27.25	Pterocephanoside A	$C_{58}H_{78}O_{30}$	1253.4529	2.9	1277.4491 [M+Na]$^+$, 1093.4158 [M+H−Glc]$^+$, 931.3617 [M+H−2Glc]$^+$
53	28.35	Sylvestroside IV dimethyl acetal	$C_{29}H_{42}O_{15}$	629.2449	0.6	653.2424 [M+Na]$^+$
54	28.68	Unknown 7	$C_{58}H_{78}O_{30}$	1253.4509	0.7	1277.4478 [M+Na]$^+$, 1255.4674 [M+H]$^+$
55	29.28	Unknown 8	$C_{50}H_{66}O_{23}$	1033.3916	−0.1	1057.2906 [M+Na]$^+$, 1035.4170 [M+H]$^+$
56	31.30	Kaempferol-3-*O*-(3″,6″-di-*O*-E-p-coumaroyl)-β-D-glucopyranoside	$C_{32}H_{28}O_{14}$	635.1404	0.5	1295.4634 [2M+Na]$^+$, 1273.3050 [2M+H]$^+$, 659.1376 [M+Na]$^+$, 637.1555 [M+H]$^+$
57	32.76	Semipapposide D	$C_{75}H_{122}O_{38}$	1629.7537	0.1	1653.7521 [M+Na]$^+$, 1631.7699 [M+H]$^+$
58	32.84	Hookeroside C	$C_{75}H_{122}O_{38}$	1629.7548	0.7	1653.7531 [M+Na]$^+$

59	32.87	Hookeroside B	$C_{69}H_{112}O_{34}$	1483.6960	0.2	
60	32.91	Hookeroside A	$C_{64}H_{104}O_{30}$	1351.6548	1.0	1029.3523 [M+H−2Glc]$^+$, 867.3119 [M+H−3Glc]$^+$
61	33.13	Unknown 9	$C_{75}H_{122}O_{38}$	1629.7556	1.2	1653.7523 [M+Na]$^+$, 1631.7703 [M+H]$^+$
62	33.26	Scoposide G	$C_{58}H_{94}O_{25}$	1189.6018	1.6	1213.5975 [M+Na]$^+$, 1191.6156 [M+H]$^+$
63	33.40	3-*O*-α-L-Rha-(1→2)-α-L-Ara Hederagenin-28-*O*-β-D-xylyl-(1→6)-β-D-Glc ester	$C_{53}H_{86}O_{21}$	1057.5599	1.5	1081.5563 [M+Na]$^+$, 1059.5515 [M+H]$^+$
64	33.99	Songoroside M-isomer	$C_{63}H_{102}O_{29}$	1321.6450	1.6	1345.6415 [M+Na]$^+$
65	34.38	*O*-β-D-Glc-(1→2)-*O*-[β-D-Xyl-(1→3)]-*O*-β-D-Xyl-(1→2)-β-D-Glc (3β)-3-[(2-*O*-β-D-Xyl-β-D-Glc)oxy]olean-12-en-28-oate	$C_{63}H_{102}O_{30}$	1337.6399	1.6	1361.6356 [M+Na]$^+$
66	34.63	Yemuoside YM_{21}	$C_{63}H_{100}O_{29}$	1319.6299	2.0	1343.6257 [M+Na]$^+$, 1321.6466 [M+H]$^+$
67	34.63	Dipsacus saponin P	$C_{58}H_{94}O_{26}$	1205.5972	1.4	1229.5935 [M+Na]$^+$
68	34.76	Scoposide E	$C_{46}H_{74}O_{15}$	865.4958	1.0	889.4929 [M+Na]$^+$, 867.3104 [M+H]$^+$
69	34.84	Virgaureasaponin B	$C_{71}H_{114}O_{33}$	1493.7185	1.4	1517.7159 [M+Na]$^+$, 1495.7324 [M+H]$^+$
70	35.10	Eupteleasaponin III	$C_{63}H_{100}O_{29}$	1319.6299	2.0	1343.6240 [M+Na]$^+$, 1321.6427 [M+H]$^+$
71	35.17	Eupteleasaponin II	$C_{58}H_{92}O_{25}$	1187.5862	1.1	1211.5829 [M+Na]$^+$, 1189.6013 [M+H]$^+$
72	36.79	Euscaphic acid	$C_{30}H_{48}O_5$	487.3411	1.6	511.3394 [M+Na]$^+$
73	38.64	Scabioside F	$C_{57}H_{92}O_{24}$	1159.5903	0.3	1183.5872 [M+Na]$^+$, 1161.6078 [M+H]$^+$, 1029.5652 [M+H−Xyl]$^+$
74	39.00	Songoroside M	$C_{63}H_{102}O_{29}$	1321.6445	1.2	1345.6409 [M+Na]$^+$, 1323.6648 [M+H]$^+$
75	39.50	Pterohoonoid C	$C_{30}H_{44}O_5$	483.3112	0.4	507.3085 [M+Na]$^+$, 485.3266 [M+H]$^+$
76	40.45	Pterohoonoid A	$C_{30}H_{44}O_5$	483.3120	2.1	507.3092 [M+Na]$^+$, 485.3271 [M+H]$^+$

77	41.05	Sapindoside G	$C_{63}H_{102}O_{28}$	1305.6498	1.5	1329.6455 [M+Na]$^+$, 1307.6644 [M+H]$^+$
78	41.50	Prosapogenin Bx	$C_{57}H_{92}O_{24}$	1159.5909	0.8	1183.5881 [M+Na]$^+$, 1161.6073 [M+H]$^+$, 1029.5668 [M+H−Xyl]$^+$
79	42.19	Sapindoside G-isomer	$C_{63}H_{102}O_{28}$	1305.6519	3.1	1329.6459 [M+Na]$^+$, 1307.6648 [M+H]$^+$
80	42.68	3-*O*-β-D-Xyl-(1 →4)-β-D-Glc-(1→4)-β-D-Xyl-(1→3)-α-L-Rha-(1→2)-α-L-Xyl oleanolic acid	$C_{57}H_{92}O_{24}$	1159.5917	1.5	1183.5869 [M+Na]$^+$, 1161.6052 [M+H]$^+$, 1029.5630 [M+H−Xyl]$^+$
81	43.18	Prosapogenin Ax	$C_{52}H_{84}O_{20}$	1027.5483	0.5	1051.5463 [M+Na]$^+$, 1029.5640 [M+H]$^+$
82	43.18	Bretschnoside A	$C_{64}H_{104}O_{29}$	1335.6599	1.0	1359.6542 [M+Na]$^+$, 1337.6755 [M+H]$^+$
83	44.05	Hookeroside D	$C_{63}H_{102}O_{28}$	1305.6519	3.1	1329.6467 [M+Na]$^+$, 1307.6646 [M+H]$^+$
84	46.17	Hookeroside D-isomer	$C_{63}H_{102}O_{28}$	1305.6486	0.5	1329.6455[M+Na]$^+$,1307.6583[M+H]$^+$
85	46.56	Pterocephin A	$C_{62}H_{100}O_{27}$	1275.6390	1.3	1299.6351 [M+Na]$^+$, 1277.6534 [M+H]$^+$, 1145.6116 [M+H−Xyl]$^+$, 999.5491 [M+H−Xyl−Rha]$^+$, 867.5114 [M+H−2Xyl−Rha]$^+$, 735.4683 [M+H−3Xyl−Rha]$^+$, 589.4104 [M+H−3Xyl−2Rha]$^+$, 457.3684 [M+H−4Xyl−2Rha]$^+$
86	47.37	Rivularicin	$C_{62}H_{100}O_{27}$	1275.6387	1.0	1299.6360 [M+Na]$^+$, 1277.6539 [M+H]$^+$, 1145.6123 [M+H−Xyl]$^+$, 1013.5699 [M+H−2Xyl]$^+$, 867.5115 [M+H−2Xyl−Rha]$^+$, 735.4688 [M+H−3Xyl−Rha]$^+$, 589.4155 [M+H−3Xyl−2Rha]$^+$, 457.3689 [M+H−4Xyl−2Rha]$^+$
87	48.53	Triploside G	$C_{52}H_{84}O_{19}$	1011.5540	1.1	1035.5502 [M+Na]$^+$, 1013.5689 [M+H]$^+$
88	48.93	Prosapogenins Ax-3	$C_{46}H_{74}O_{15}$	865.4960	1.3	889.4931 [M+Na]$^+$, 867.5114 [M+H]$^+$
89	51.30	Pterohoonoid D	$C_{30}H_{46}O_4$	469.3322	0.9	471.3478 [M+H]$^+$
90	51.42	Giganteaside D	$C_{41}H_{66}O_{11}$	733.4524	−0.4	1469.9717 [2M+H]$^+$, 757.4506 [M+Na]$^+$, 735.4669 [M+H]$^+$
91	52.29	11-Oxooleanolic acid	$C_{30}H_{46}O_4$	469.3318	0.0	471.3477 [M+H]$^+$
92	53.00	Songoroside A-isomer	$C_{35}H_{56}O_7$	587.3945	−0.5	611.3918 [M+Na]$^+$, 457.3685 [M+H−Xyl]$^+$
93	53.12	Schekwangsienin	$C_{30}H_{44}O_4$	467.3174	2.8	469.3320 [M+H]$^+$

94	53.27	Pterohoonoid B	$C_{30}H_{42}O_4$	465.3006	0.2	933.7078 $[2M+H]^+$, 467.3163 $[M+H]^+$
95	53.89	Songoroside A	$C_{35}H_{56}O_7$	587.3944	−0.7	611.3919 $[M+Na]^+$, 457.3681 $[M+H-Xyl]^+$
96	54.56	Atricin A	$C_{30}H_{46}O_4$	469.3328	2.1	941.6898 $[2M+H]^+$, 493.3291 $[M+Na]^+$, 471.3474 $[M+H]^+$
97	55.06	Schekwangsienin-isomer	$C_{30}H_{44}O_4$	467.3169	1.7	959.6380 $[2M+Na]^+$, 937.6564 $[2M+H]^+$, 469.3318 $[M+H]^+$
98	56.66	Hookeroside D-isomer	$C_{63}H_{102}O_{28}$	1305.6503	1.8	1329.6454 $[M+Na]^+$, 1307.6669 $[M+H]^+$
99	57.62	Oleanolic acid	$C_{30}H_{48}O_3$	455.3532	1.5	
100	57.67	Ursolic acid	$C_{30}H_{48}O_3$	455.3533	1.8	
101	58.24	Oleanonic acid	$C_{30}H_{46}O_3$	453.3386	3.7	909.6978 $[2M+H]^+$, 455.3533$[M+H]^+$, 437.3430 $[M+H-H_2O]^+$, 409.3467 $[M+H-H_2O-CO]^+$, 391.3361 $[M+H-H_2O-CO-H_2O]^+$

Note: Glc=glucopyranosyl, Rha=mannopyranosyl, Xyl=xylopyranosyl, Ara=arabinopyranosy
